# Study on the Wetting and Permeation Properties of Bio-Oil as Bitumen Rejuvenator

**DOI:** 10.3390/ijms24076512

**Published:** 2023-03-30

**Authors:** Xuewen Zheng, Wenyuan Xu, Weishuai Ji, Kai Cao

**Affiliations:** 1 School of Civil Engineering, Northeast Forestry University, Harbin 150040, China; 2 College of Civil Engineering and Architecture, Zhejiang University, Hangzhou 310058, China

**Keywords:** bio-oil, rejuvenator, aged bitumen, wettability, permeability, diffusion

## Abstract

In order to explore the diffusion and regeneration of bio-oil in aged bitumen, waste cooking oil (WCO), waste wood oil (WWO) and straw liquefied residue oil (SLRO) were selected in this paper. According to the surface wetting theory, the contact angle is obtained by combining laboratory experiments with molecular dynamics (MD) simulation, and the wetting parameters are calculated to evaluate the wetting behavior of bio-oil. The experimental phenomena of the wetting process and the main factors driving wetting are further analyzed. A permeation experiment is designed to obtain the permeation fusion layer (PFL). If the crossover modulus of PFLs changes compared with that of the aged bitumen, it is determined that the bio-oil penetrates the corresponding fusion layer. The results show that the motion of bio-oil included spreading and shrinking processes, and a precursor film played a pivotal role in the transportation of nanodroplets. Higher surface tension, lower viscosity and cohesion can effectively promote the wettability of bio-oil. A higher temperature and a longer permeation time are conducive to the permeation of bio-oil in aged bitumen. WCO with the strongest wettability has the weakest permeability, while WWO has superior permeability and can activate the macromolecules’ surface activity, but its wettability is relatively weak. It is necessary to further modify WCO and WWO to be suitable rejuvenators.

## 1. Introduction

With the development of transportation industry, a large part of asphalt pavement is faced with maintenance and even reconstruction, which needs maintenance to extend its service life. Although preventive maintenance and other technical measures can increase the service life of asphalt pavement to a certain extent, simple maintenance cannot repair structural damage to pavements. Therefore, the pavement needs to be overhauled or even rebuilt every few years, resulting in a large amount of waste asphalt mixture every year. The reclaimed asphalt pavement (RAP) can not only solve the urgent needs of highway construction materials, but can also save oil resources and promote the green and sustainable development of highway construction [1]. In the recycling technology of old asphalt mixture, a key problem is the mixing of new and old asphalt. The mixing degree directly affects the road performance of the recycled mixture [2]. The mixing process of new and old asphalt mixture mainly depends on the friction between aggregates. In fact, this friction is a shear action. In the ideal state, this shear motion pattern is not conducive to the mutual fusion of layered bitumen on the aggregate surface, especially at low mixing temperature, due to the large difference in viscosity between them. At the same time, the coarse surface of aggregate also makes the composition exchange between new and old bitumen more unfavorable. In addition, the dust and fine aggregate on the aggregate surface are among the main factors hindering the exchange of new and old bitumen components. Thus, it is difficult to reduce the bitumen film unevenness on the aggregate surface through the mixing process. The improvement of this non-uniformity mainly depends on the interaction between fresh bitumen and aged bitumen or aged bitumen and rejuvenator. This is where the rejuvenator is crucial. In the process of mixture mixing, construction and service, the phenomenon of fusion and diffusion between each layer of bitumen and rejuvenator takes place until full fusion. The mechanical properties of materials before and after fusion are completely different, and their mechanical responses are different in different states. When the rejuvenator is completely fused with the aged bitumen, the rejuvenated bitumen will obtain the best performance. There is no doubt that the degree of diffusion and fusion between the old and new bitumen determines the overall performance of the recycled mixture. Therefore, the rejuvenator not only needs to have the ability to restore the properties of aged bitumen, but more importantly, it needs to have the ability of rapid diffusion and fusion. It can be seen that analyzing the diffusion characteristics of different rejuvenators and identifying the rejuvenators with fast diffusion can lay a solid foundation for further narrowing the screening range of raw materials.

The research methods of rejuvenator diffusion are mainly laboratory tests, molecular dynamics (MD) simulation and model prediction. Qi et al. put the aged bitumen softening point sample into the rejuvenator and analyzed the diffusion degree by measuring the change in the softening point in different environments. Based on the splitting strength test, the influence of the diffusion degree of the rejuvenator on the performance of RAP was analyzed [3]. Ding et al. used atomic force microscopy (AFM), Fourier transform infrared spectroscopy (FTIR) and a dynamic shear rheometer (DSR) to study the changes in various indicators before and after the fusion of new and old bitumen [4]. Fang et al. prepared the recycled fusion layer (RFL) of rejuvenators and aged bitumen using a permeation test. Then, after measuring the viscosity of the RFLs, several factors affecting the viscosity of the RFLs were discussed. Combined with Fick’s law and composite material theory, the permeability coefficient of rejuvenators in aged bitumen was calculated [5]. Subsequently, their team compared the wetting behavior of different rejuvenators on the aged bitumen surface based on the surface wetting theory and systematically analyzed the factors affecting the wetting behavior [6]. Xiao et al. used the contact angle between the rejuvenator and the aged bitumen surface, interface image threshold analysis and GC-MS oil tracer analysis to characterize the diffusion of the rejuvenator in aged bitumen [7]. Xu designed a diffusion experiment based on viscosity and a SARA test to evaluate the diffusion rate of rejuvenator [8]. Wei et al. proposed using layered extraction technology, gel permeation chromatography (GPC), FTIR, scanning electron microscope (SEM), energy dispersive spectrometer (EDS) and AFM to study the diffusion process of fresh bitumen/rejuvenator in the aged bitumen layer and characterize the degree of miscibility between fresh bitumen/rejuvenator and aged bitumen, as well as the regeneration effect [9]. Ma et al. designed a diffusion experiment on the basis of a permeation test and a DSR test to evaluate the diffusion degree of the rejuvenator in aged bitumen [10]. Uwe et al. predicted the influence of SARA composition and aging stage on spurt completion time and diffusivity in bitumen through diffusion-reaction modeling [11]. MD simulation is mainly used to study the rejuvenator diffusion by establishing a layered model, analyzing the influence of temperature and rejuvenator type on the rejuvenator diffusion, and quantifying the rejuvenator diffusion by calculating the mean square displacement and relative concentration distribution [12,13,14,15,16,17,18].

The existing studies above have made fruitful achievements in the evaluation methods, mechanisms, models and factors affecting the rejuvenator diffusion, but the relevant research is based on the fact that the rejuvenator has completely wrapped around the aged bitumen surface. However, according to the material surface theory, when the rejuvenator is mixed with RAP particles, the rejuvenator first diffuses on the surface of RAP particles, and then permeates into the old bitumen, thus restoring the colloidal structure and performance of the old bitumen. It can be inferred that the full spreading of the rejuvenator on the surface of RAP particles is an antecedent factor to ensure its full permeation into the aged bitumen. If the interface wettability between the rejuvenator and the old bitumen is poor, it is difficult for the rejuvenator to fully wrap the RAP particles, which is not conducive to the interface permeation between the rejuvenator and the old bitumen and will adversely affect the performance of the recycled asphalt mixture. A lot of studies have been conducted to study the interface diffusion behavior and influencing factors between rejuvenator and aged bitumen by surface wetting theory [6,19,20,21]. However, these studies were limited in evaluating the diffusivity of rejuvenators. In fact, wettability and permeability should be considered comprehensively when considering the diffusion properties of rejuvenators. In this paper, the wettability and permeability of rejuvenator are comprehensively explored by combining laboratory tests and MD simulation, and the motion phenomenon and driving mechanism of rejuvenator during the wetting process are further supplemented. In addition, the existing research on the wetting behavior of rejuvenator on the aged bitumen surface generally adopts the laboratory test method, and there is less research on the application of MD simulation to evaluate the wetting performance, but MD can deeply explain the wetting mechanism of driving droplets, which is the main reason why MD is selected as the research method in this paper.

At present, bio-oil is receiving extensive attention as a sustainable bitumen regeneration modifier. The source comes from pig manure [16,22,23,24], waste cooking oil [25,26,27,28], cashew nuts [20,29], castor oil [30,31], straw [32,33] sawdust [34,35], and so on. Previous studies have not fully analyzed the diffusion performance of bio-oil and rarely mentioned the wettability of bio-oil, which means that there is still insufficient reason for these bio-oils to be suitable as aged bitumen regeneration modifiers. Hence, three kinds of bio-oils were selected as the rejuvenator of aged bitumen in this paper, namely waste cooking oil (WCO), waste wood oil (WWO) and straw liquefied residue oil (SLRO). Selecting bio-oil as the research object can not only reveal the common phenomenon of rejuvenator diffusion performance, but can also effectively identify whether a bio-oil is a qualified rejuvenator, thus providing scientific theoretical support for road construction and regenerant research and development. In the research, firstly, the wetting behavior of bio-oil droplets on the aged bitumen surface was investigated and the influencing factors were analyzed. Secondly, the permeability of bio-oil was studied and the main factors affecting the permeability were analyzed.

## 2. Results and Discussion

### 2.1. Contact Angles Analysis

In the MD simulation, the wettability of three bio-oils on bitumen surfaces with different aging degrees and the influence of different temperatures on the wettability were simulated.

**Figure 1:**
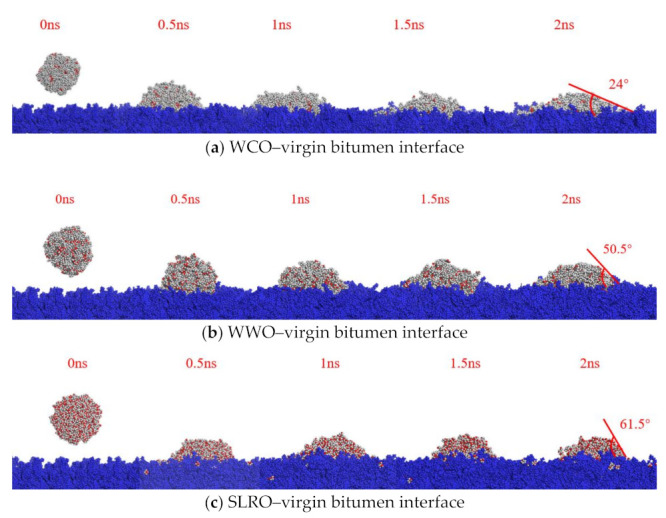
Wetting process of bio-oils nano-droplets on virgin bitumen surface at 408.15 K.

Figure 1 shows the corresponding typical equilibrium snapshots of bio-oils nanodroplets spreading on the virgin bitumen surface, taking the simulation temperature at 408.15 K as an example. During spreading in the MD simulation, bio-oils nanodroplets move downward and spread to the bitumen surface in simulations. However, the wetting rate and degree on the bitumen surface are obviously different. Moreover, the wetting behavior of the three bio-oils on the surface of virgin bitumen basically reaches a stable state after 1.5 ns. Compared with WWO and SLRO, the spreading of WCO on the surface of virgin bitumen is obviously faster and more affine. WWO is the second and SLRO is the weakest. After the wetting behavior is stable, the contact angles of the three bio-oils on the virgin bitumen surface are SLRO > WWO > WCO, and the values are less than 90°. This shows that the wetting process of the three bio-oils on the virgin bitumen surface can be carried out spontaneously. However, WCO is easier to wet on the virgin bitumen surface than the other two bio-oils. When all simulated systems are balanced at 408.15 K, the contact angle between the bio-oils and different bitumen surfaces in various simulation scenarios is measured. Meanwhile, in order to verify the accuracy of the simulation results, the contact angles between the three bio-oils and the bitumen surfaces are measured in laboratory experiments for comparison with the simulation results, as shown in Table 1.

As can be seen in Table 1, the development trend of the contact angles between the three bio-oils and different bitumen surfaces is consistent, and the difference between the contact angle measured by MD and laboratory test is less than 3°, indicating that the contact angle between the bio-oils and bitumen surfaces simulated by MD is reasonable.

#### 2.1.1. Analysis of the Influence of Different Aging Degrees on the Interface Contact Angles between Bio-Oils and Bitumen

**Figure 2:**
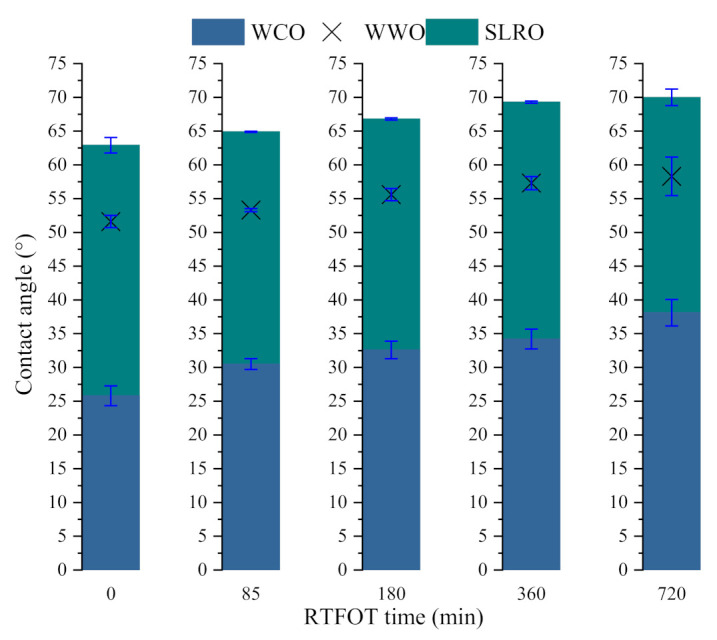
Contact angle between bio-oils and different aged bitumen surfaces at 408.15 K.

The contact angles between bio-oils and different aged bitumen surfaces are shown in Figure 2. The trend of contact angle curves is basically consistent, and all values are less than 90°, indicating that three bio-oils can spontaneously wet bitumen surfaces at different aging degrees. Furthermore, no matter how the bitumen surface is replaced, the contact angle formed by WCO on the bitumen surface is the smallest. This demonstrates that WCO is easier to wet on the bitumen surface than other two oils, followed by WWO and SLRO. On the other hand, the contact angle of the three oils on the bitumen surface increases with the severity of aging. This shows that aging will hinder the spread of bio-oils in the horizontal direction, reduce the wetting effect of bio-oils and affect the regeneration consequences. This is because aging treatment will re-arrange the surface atoms and lead to reduced surface energy and interaction with bio-oils [36]. Simultaneously, the bio-oils are more significant to the surface wetting of the aged bitumen within three hours, while the difference of contact angle formed on the surface between RTFOT 360 min and RTFOT 720 min is small, and the wetting behavior of WCO is more sensitive to the aging degree than the other two bio-oils.

#### 2.1.2. Analysis of the Influence of Temperature on the Interface Contact Angles between Bio-Oils and Bitumen

**Figure 3:**
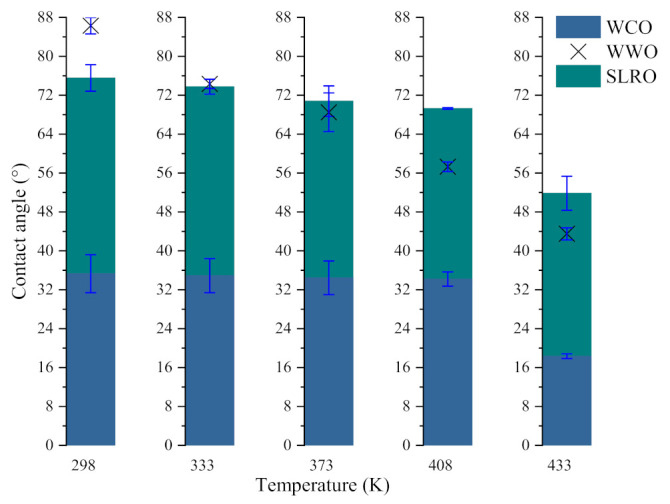
Bio-oils/RTFOT 360 min surfaces contact angles at different temperatures.

In order to analyze the influence of different temperatures on the wetting behavior of bio-oils, taking the bio-oils/RTFOT 360 min interface as an example, the contact angles formed by three bio-oils on the RTFOT 360 min surface at different temperatures are measured. The results are shown in Figure 3.

As can be seen in Figure 3, with the increase in temperature, the contact angles formed by bio-oils/RTFOT 360 min show a downward trend. The higher the temperature, the lower the value, and all the values are lower than 90°, indicating that the increase in temperature is conducive to the three bio-oils spreading on the surface of aged bitumen, making it easier to proceed with spontaneous wetting behavior. However, the contact angles produced by several oils decrease at different rates with the temperature. The contact angle created by WWO decreases the fastest with the increase in temperature, basically in a straight line, while the contact angles created by WCO and SLRO do not decrease significantly before 408.15 K. This is especially true of WCO. This indicates that the wetting of WWO is more sensitive to temperature. When the temperature is between 408.15 K–433.15 K, the WCO/RTFOT 360 min and SLRO/RTFOT 360 min contact angles decrease significantly, indicating that this temperature range is more favorable for the wetting of WCO and SLRO. At the same time, it can be seen that at any temperature, the wetting contact angle of WCO is the smallest of the three bio-oils, indicating that WCO has the best expansibility on the aged bitumen surface. Even at 433.15 K, WWO and SLRO are not as scalable as WCO. It should be noted that before 333.15 K, the contact angle of WWO is greater than SLRO, and after this temperature, the contact angle is less than SLRO, indicating that the wettability of WWO is better after 333.15 K.

### 2.2. Effects of Adhesion Work and Infiltration Work on Wetting Behavior

**Figure 4:**
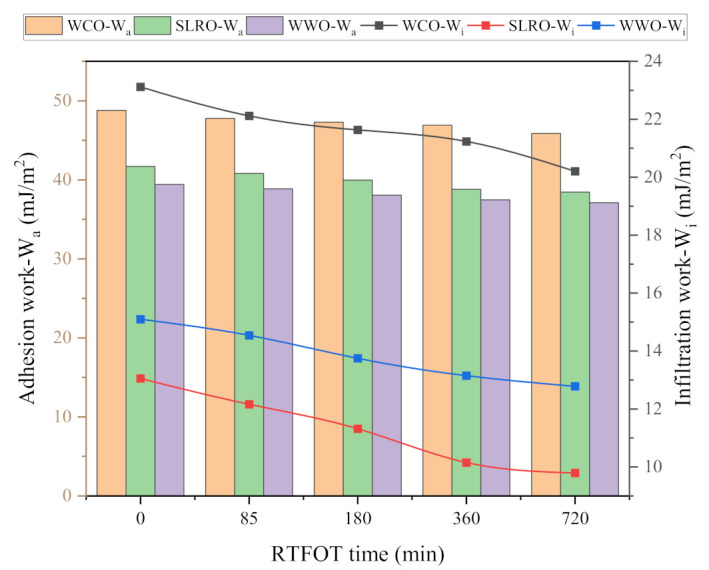
W_a_ and W_i_ of the bio-oils on different aged bitumen surfaces at 408.15 K.

**Figure 5:**
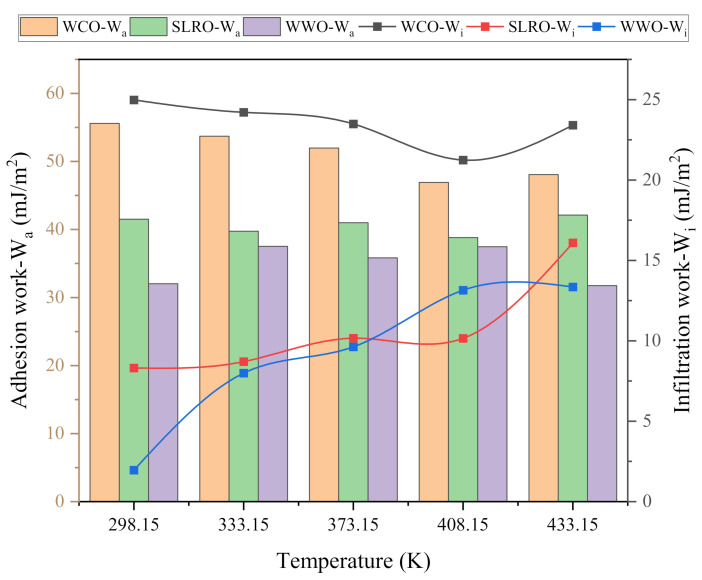
W_a_ and W_i_ of the bio-oils on RTFOT 360 min surface at different temperatures.

According to the above analysis, the contact angles between the three bio-oils and bitumen are less than 90°, indicating that the wetting behavior can be carried out spontaneously. In order to further evaluate the diffusion performance of the bio-oils, the aging degree of bitumen at 408.15 K and different temperatures on the RTFOT 360 min surface are taken as variables. The adhesion work W_a_ and infiltration work W_i_ are calculated according to Formulas (6) and (7). The calculated results are shown in Figure 4 and Figure 5. W_a_ indicates the firmness of solid–liquid two-phase spreading and bonding, and W_i_ implies the difficulty of the infiltration process. As can be seen from Figure 4, with the increase in the aging time, the W_a_ and W_i_ of the bio-oils on the bitumen surface gradually decrease, indicating aging can weaken the spread bond of bio-oils on the bitumen surface and limit the motility of infiltration. Therefore, the wettability of bio-oils to bitumen is further restricted. The more serious the aging, the more difficult the wetting of the bio-oils. This may be related to the surface free energy of bitumen: aging will reduce the surface free energy of bitumen [37], weaken the adhesion of bitumen and then affect the adhesion between bio-oils and bitumen. In addition, according to the compatibility theory, after bitumen aging, the content of asphaltene increases and the light components decrease, which makes the proportion of maltene and asphaltene unbalanced, aggravates the poor solubility parameters between asphaltene and maltene and thus reduces the compatibility and adhesion of bitumen. On the other hand, the interaction between molecules increases and the free volume decreases after aging, making infiltration more difficult. It can also be seen from Figure 4 that on any aged bitumen surface, W_a_ and W_i_ of WCO are the largest among the three bio-oils, indicating that WCO has the strongest spreading bond and the easiest infiltration on the bitumen surface. However, for WWO and SLRO, the results of W_a_ and W_i_ are inconsistent. For W_a_, SLRO is more significant, while for W_i_, WWO is superior. From Formulas (6) and (7), it can be seen that W_a_ and W_i_ are closely related to the surface tension and contact angle of the bio-oils. The comparison of W_a_ and W_i_ of SLRO and WWO depends on which parameter of surface tension and contact angle is more prominent. For W_a_, since the surface tension of SLRO is greater than that of WWO, the surface tension determines that the W_a_ of SLRO is better than that of WWO, and the effect of the contact angle is not as strong as the surface tension. For W_i_, the contact angle plays a stronger decisive role. Since the contact angle of WWO is significantly smaller than that of SLRO, the W_i_ of WWO is larger than that of SLRO.

The effect of temperature on W_a_ and W_i_ of the three bio-oils is obviously different from that of aging. As can be seen in Figure 5, the W_a_ of WCO on bitumen surface gradually decreases with the increase in temperature, but the W_a_ at 433.15 K is greater than that at 408.15 K. This shows that increasing the temperature will weaken the spreading and bonding of WCO on the bitumen surface. This is because the increase in temperature will reduce the surface tension of WCO, and the contact angle between WCO and bitumen does not decrease significantly with the increase in temperature. In addition, because the contact angle of WCO-bitumen interface decreases significantly at 408.15 K, the W_a_ increases. The effect of temperature on the W_a_ of WWO and SLRO has no obvious rule, as shown by different decreasing degrees of surface tension and contact angle. In addition, the W_a_ of WCO on the bitumen surface at different temperatures is greater than that of WWO and SLRO, indicating that the wettability of WCO on aged bitumen at different temperatures is better than that of WWO and SLRO. Concurrently, the W_a_ of the three bio-oils have a large difference at low temperature. When the temperature increases, this gap gradually decreases, especially for WCO. On the other hand, similar to the W_a_, the W_i_ of WCO on the bitumen surface gradually decreases with the increase in temperature and increases at 433.15 K. The reason is similar to that of the W_a_ and will not be repeated here. Unlike WCO, the W_i_ of WWO and SLRO gradually increases with the increase in temperature, and the difference between them grows larger with the increase in temperature. The greater the surface tension, the smaller the contact angle and the greater the W_i_. Since the surface tension difference between WWO and SLRO is small, the W_i_ mainly depends on the contact angle. Before 373.15 K, the contact angle of WWO on the bitumen surface is greater than that of SLRO, so the W_i_ of SLRO is greater than that of WWO. When the temperature exceeds 373.15 K and is less than 433.15 K, the contact angle formed by WWO is less than that of SLRO, so the W_i_ of WWO in this temperature range is greater than that of SLRO. When the temperature is 433.15 K, the reduction in the contact angle formed by WWO is less than that of the surface tension, so the W_i_ of WWO is less than that of SLRO. In addition, the W_i_ of WCO varies slightly with the temperature, which means that its wetting ability is similar at high and low temperatures. The W_i_ of WWO tends to stabilize after 408.15 K, while the W_i_ of SLRO increases rapidly after 408.15 K. Therefore, when WWO and SLRO are applied to bitumen regeneration, the wetting ability is best when the mixing temperature is no lower than 408.15 K.

### 2.3. Analysis of Wetting Rate and Time of Bio-Oils

**Figure 6:**
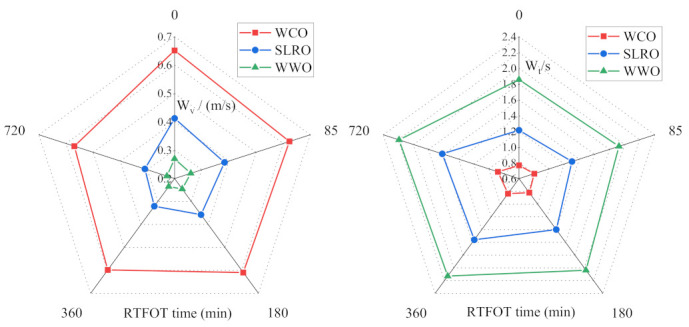
W_v_ and W_t_ varies with aging degree.

**Figure 7:**
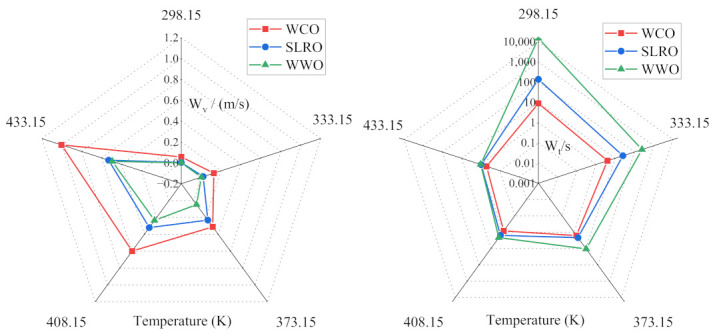
W_v_ and W_t_ varies with temperature at the interface of bio-oils-RTFOT 360 min.

Several static parameters (contact angle, W_a_, W_i_) were used to evaluate the wetting behavior of the three bio-oils at the bitumen interface. However, the wetting of rejuvenators on the surface of aged bitumen is a dynamic process. According to the surface energy theory, these dynamic parameters can be well characterized by wetting rate (W_v_) and wetting time (W_t_). The W_v_ and W_t_ of the three bio-oils are calculated according to Formulas (9) and (10). The calculation results are shown in Figure 6 and Figure 7. It can be seen from Figure 6 and Figure 7 that the greater the wetting rate, the shorter the wetting time, which means that the bio-oils cover the old bitumen faster in the actual mixing process, which is more conducive to the diffusion and permeation of bio-oils and old bitumen.

In Figure 6, with the severity of aging, the wetting rate of bio-oils decreases gradually, and the corresponding wetting time increases gradually, indicating that aging will reduce the coating speed of bio-oils on the surface of old bitumen. WCO has the fastest W_v_ and the shortest W_t_ among all bio-oils, followed by SLRO and WWO. This is mainly determined by the viscosity of the three bio-oils. The viscosity order of the three bio-oils is WCO < SLRO < WWO, which leads to the wetting speed WCO > SLRO > WWO. At this point, the influence of surface tension and contact angle is not significant compared with the viscosity.

In Figure 7, temperature has a significant effect on the wetting rate of bio-oils. With the increase in temperature, the W_v_ of bio-oils increases and the W_t_ decreases gradually. The wetting rate of WWO increases most obviously with the increase in temperature. It shows that increasing the temperature is conducive to the rapid coating of old bitumen with bio-oils, and the wetting rate of WWO is greatly affected by temperature. In addition, before 373.15 K, the difference of W_v_ and W_t_ between the three bio-oils is obvious. For example, at 298.15 K, the W_t_ of WWO is 107 times that of SLRO and 1668 times that of WCO. When the temperature is higher than 373.15 K, the gap between them decreases gradually. The wetting rate of WCO is the fastest at any temperature, and the change with temperature is far less obvious than that of SLRO and WWO. Even at low temperatures, the wetting rate is still faster. The difference in the wetting rate is still determined by the difference in viscosity.

### 2.4. Effect of Viscosity and Surface Tension of Bio-Oil on Wettability

**Figure 8:**
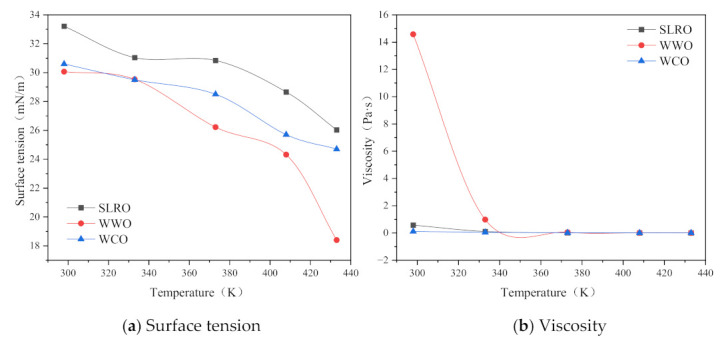
Surface tension and viscosity of bio-oil vary with temperature.

The above analysis studies the influence of aging degree and temperature on the wettability of bio-oils. This section studies the effects of viscosity and surface tension of bio-oil on its wettability. Before studying the influence of viscosity and surface tension on wettability, the changes in the viscosity and surface tension of three kinds of bio-oil with the changes in temperature were first analyzed, as shown in Figure 8.

Figure 8 shows that surface tension and viscosity decrease with the increase in temperature. Among the three bio-oils, WCO has the highest surface tension and the lowest viscosity. WWO has the lowest surface tension and the highest viscosity. When the temperature is less than 333.15 K, the viscosity of WWO is much higher than that of WCO and SLRO, while the surface tension of SLRO and WWO is similar. According to the above analysis, the wettability of SLRO is superior to that of WWO, which indicates that the influence of viscosity on the wettability of bio-oil is greater than that of surface tension.

#### 2.4.1. Influence of Viscosity on Wetting Performance

**Figure 9:**
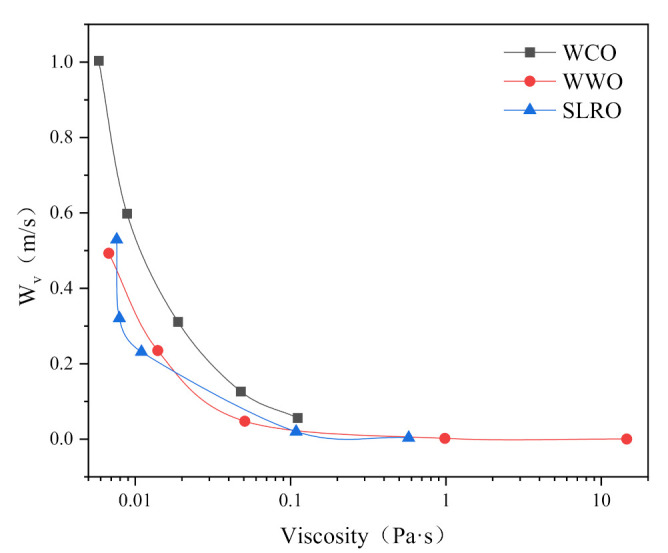
Effect of viscosity on W_v_.

The viscosity is closely related to fluidity. The higher the viscosity, the poorer the fluidity. Therefore, the wettability of bio-oil on the aged bitumen surface is closely related to its viscosity. According to Formulas (7) and (9), viscosity has no correlation with W_i_, but W_v_ is closely related to viscosity. Thus, this section analyzes the effect of viscosity on the W_v_ of bio-oil. The results are shown in Figure 9. As can be seen from Figure 9, with the increase in viscosity, the wetting velocity of bio-oil gradually decreases and finally approaches 0, which shows that viscosity has a significant effect on the wettability of bio-oil on the aged bitumen surface. However, when the viscosity of bio-oil increases to a certain extent, the W_v_ decreases to 0. By comparing the viscosity-W_v_ curves of the three bio-oil, it can be seen that the wettability of WCO is higher than that of WWO and SLRO, indicating that WCO has stronger wettability than WWO and SLRO. When the viscosity is less than 1 Pa·s, the W_v_ of WWO and SLRO decreases at similar rates, indicating that their wettability on aged bitumen is basically the same when the viscosity is less than 1 Pa·s. In addition, according to the previous analysis, it is known that WCO has the strongest wettability. Combined with the results of Figure 8, it can be concluded that the larger the surface tension, and the smaller the viscosity and contact angle, the better the wettability of the rejuvenator. Therefore, when selecting rejuvenators, factors such as high surface tension, low viscosity and small contact angle should be fully considered. In particular, low viscosity is the primary consideration.

#### 2.4.2. Influence of Surface Tension on Wetting Performance

**Figure 10:**
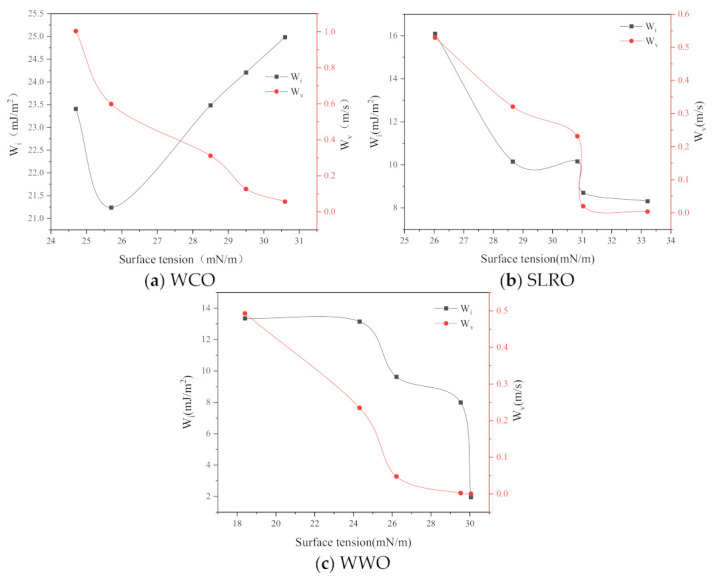
Influence of surface tension on wetting.

At the boundary between liquid and gas, i.e., the liquid surface and the interface between two immiscible liquids, an extremely small pulling force is generated due to the attraction between molecules. Suppose there is a thin film layer on the surface, which bears the tensile force of the surface. This tensile force of the liquid is called the surface tension, which results in the reduction of the liquid surface. Thus, for rejuvenator droplets, the greater the surface tension of the rejuvenators, the greater the effort required to reduce its surface, and the easier the wetting process between it and the aged bitumen. Therefore, surface tension is one of the most important factors affecting the wettability of aged bitumen by rejuvenators. Figure 10 shows the changes in W_i_ and W_v_ with the surface tension during wetting process. As can be seen from Figure 10, the W_i_ of WCO decreases first and then increases with the surface tension. The W_v_ and W_i_ of the three bio-oils decrease with the increase in surface tension, indicating that the higher the surface tension, the worse the wetting ability of bio-oil, which is inconsistent with previous research [19]. The reason for this phenomenon is that the surface tension and viscosity have the same trend with temperature. Figure 8 shows that as the surface tension increases, so does the viscosity. The increase in surface tension leads to the increase in W_i_, while the increase in viscosity leads to the decrease in wettability. The influence of viscosity is greater than that of surface tension. Therefore, the wettability of bio-oil on the surface of aged bitumen decreases. In addition, the reason why the W_i_ of WCO decreases first and then increases with the surface tension is that the contact angle increases greatly at the turning point. In summary, the wettability of rejuvenator is affected by surface tension, viscosity and contact angle. When other factors remain unchanged, the higher the surface tension of the rejuvenator, the better the wettability.

### 2.5. Wetting Mechanism Analysis of Bio-Oil

#### 2.5.1. The Motion of Bio-Oils Droplets on the Bitumen Surface

**Figure 11:**
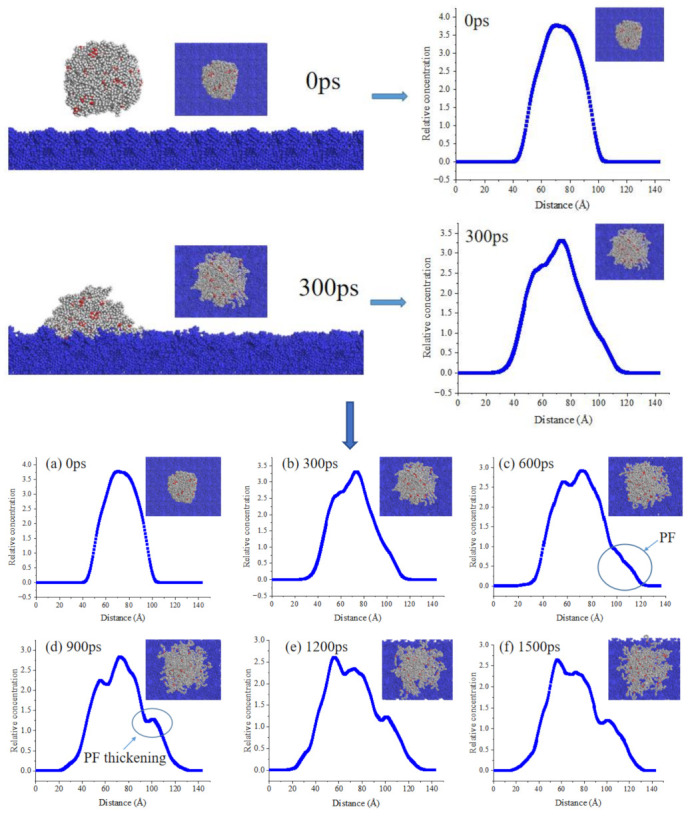
Side and top views of WCO droplet structure evolution. Note: blue block represents bitumen subgrade, blue line represents WCO droplet concentration profile.

**Figure 12:**
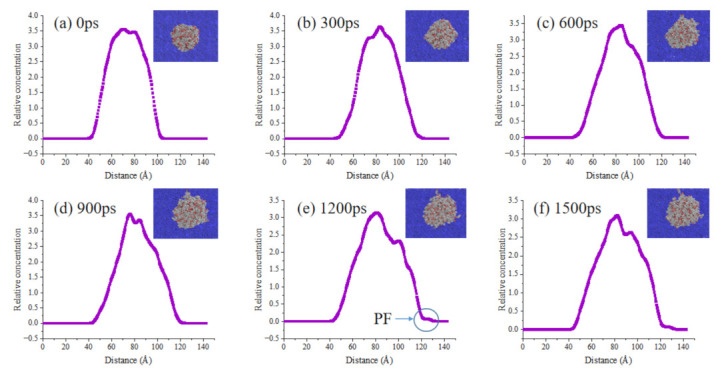
Side and top views of WWO droplet structure evolution. Note: blue block represents bitumen subgrade, purple line represents WWO droplet concentration profile.

**Figure 13:**
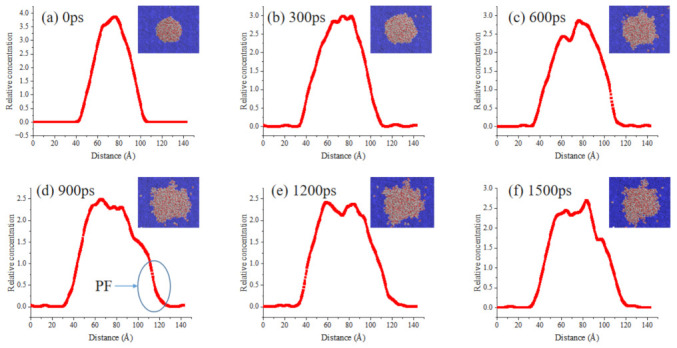
Side and top views of SLRO droplet structure evolution. Note: blue block represents bitumen subgrade, red line represents SLRO droplet concentration profile.

Figure 11, Figure 12 and Figure 13 show adsorption snapshots for bio-oil droplets at different simulation time. The initial shape (0 ps) of the three bio-oil droplets is spherical. Then, the droplets adsorb to the surface and quickly reshape to form a hemisphere (such as 300 ps in Figure 11). Meanwhile, the bio-oil droplets spread on the bitumen surface. It can be seen from Figure 11 that the side view concentration profile of the droplet can visually describe the dynamic changes of molecules during the spreading process. Therefore, the behavior of bio-oils during wetting can be described according to the side view change of molecular concentration. During the process of droplet spreading, we find that some surface molecules of bio-oils preferentially form a layer of droplet film on the bitumen surface, but this layer of film is not obvious for some bio-oils. The reason for the formation of thin films is that compared with internal molecules, these surface bio-oil molecules are unstable and active due to their unsaturated electrostatic and hydrogen bond interactions [38]. Therefore, the strong attraction between these surface molecules and the precursor surface of bitumen leads to preferential adsorption and the formation of precursor film (PF). Generally, PF is a thin film with limited thickness, which propagates in front of the droplet contact line and controls the wetting behavior [39]. Many theoretical and experimental studies on spreading droplets have confirmed the existence of PF [38,40].

By observing the structural evolution of PF in detail, a phenomenon called “spreading of bio-oil molecules on PF” is observed. As can be seen from Figure 11, with the extension of time, WCO molecules gradually spread to both sides after contacting the bitumen surface. The peak value of the highest concentration decreases gradually. In the side view, the slope of the concentration line at 0 ps is steep. With the spreading, the slope decreases to varying degrees; this is caused by the gradual movement of molecules in PF to the outside. The concentration line at the minimum of the outermost slope is regarded as PF (Figure 11c). Furthermore, it can be observed that PF is a membrane with a slope, which makes it easier for the droplet molecules behind PF to diffuse. That is, the molecules behind PF can slide down the slope. Then, the WCO molecules on the surface of the droplet escape from the droplet and slide down step by step, just like a ball rolling down a slope. In this way, the WCO molecules on the droplet surface continuously migrate from the top of the slope to the upper part of PF. Finally, these WCO molecules enter the PF and become part of it, which leads to an increase in the thickness of the PF. The platform within the contour line circled in Figure 11d is the PF thickening process. After that, the WCO droplet continues to move forward, and the molecules at the back of droplet move forward sequentially until the PF no longer changes. This leads to the shrinking of the WCO droplet, and the system gradually becomes stable. We can verify this from Figure 11e,f. Although the concentration changes in the range of 1200–1500 ps, the change is small because the droplets gradually shrink.

It can be seen from the above analysis that the faster PF is formed, the faster the bitumen is wetted by the bio-oils. Figure 12 and Figure 13 show the droplets concentration changes of WWO and SLRO during the spreading process. It can be seen that the PF formation rate of WWO and SLRO is significantly lower than that of WCO, and the obvious time of occurrence is 1200 ps and 900 ps, respectively. However, this does not mean that there is no diffusion before this time point. In this paper, an obvious time point is selected for more intuitive observation. The PF formation time sequence of the three bio-oils is WCO < SLRO < WWO, indicating that WCO has a faster wetting rate. This is consistent with the order of wetting rates of the three in Section 2.3.

#### 2.5.2. Analysis of Wetting Mechanism of Bio-Oils on Bitumen Surface

**Figure 14:**
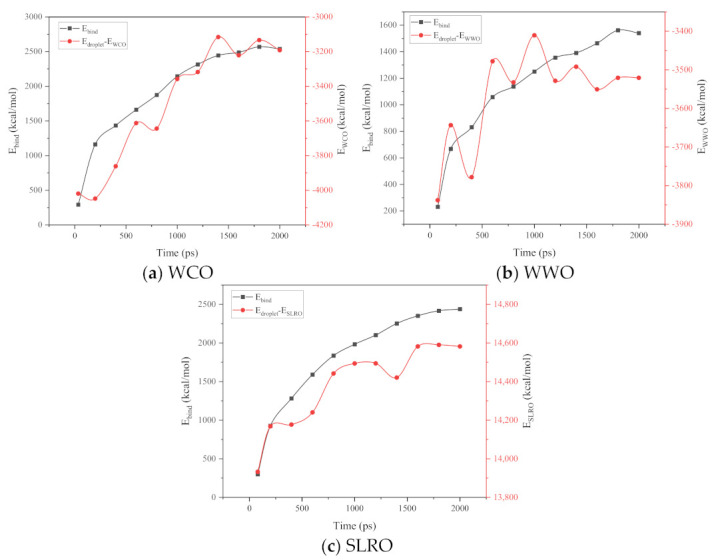
The nonbond interaction energy between the bio-oils droplets and aged bitumen surface, $E_{bind}$ and the energy of the bio-oils droplets $E_{droplet}$.

If the rejuvenator droplets want to wet the bitumen surface, they need enough driving force to overcome the resistance. In order to wet the bitumen surface, the regenerant droplets also need enough driving force to overcome the resistance. Therefore, analyzing the energy change in the wetting process is an effective means of understanding the wetting mechanism of droplets. The total energy of the wetting system can be calculated by Equation (1):

(1) $$E_{bind}=\left(E_{droplet}+E_{surface}\right)-E_{total}$$

where $E_{bind}$ represents the nonbond interaction energy between the rejuvenator droplets and the bitumen surface, $E_{droplet}$ represents the rejuvenator droplets energy, and $E_{surface}$ represents the aged bitumen surface energy. During the simulation process, the energy of bitumen surface is almost constant because the atomic vibration of the bitumen surface is very light. Therefore, according to Formula (1), $E_{droplet}$ and $E_{bind}$ determine the wetting behavior of rejuvenator droplets. The results of $E_{droplet}$ and $E_{bind}$ are shown in Figure 14. From Figure 14, with the progress of wetting process, the $E_{bind}$ of the three wetting systems gradually increases and finally tends to be stable. This is because, with the continuous spread of bio-oil molecules, the contact area between bio-oil molecules and bitumen surface increases, and the nonbond interaction between bio-oil molecules and bitumen surface molecules is enhanced. With the gradual stabilization of the droplets’ movement, the $E_{bind}$ tends to be stable. Concurrently, $E_{droplet}$ also increases gradually with the simulation, and finally tends to be stable. This is because the initial spherical droplet is the most stable and the energy is the least stable. When the droplet shape is difficult to keep spherical, the energy will change [40]. With the simulation, the three bio-oil droplets begin to spread on the bitumen substrate, and the droplets are no longer spherical under the influence of bitumen surface molecules. The increasing contact area induces the growth of $E_{droplet}$. When the droplet no longer spreads, the energy tends to be stable. In addition, comparing the energy changes of the three wetting systems, we find that the $E_{bind}$ and $E_{droplet}$ of the bio-oils with good spreading ability has a larger change rate. It can be seen from Figure 14 that among the three regenerant wetting systems, the system containing WCO has the largest energy change rate, followed by SLRO and finally WWO. This is consistent with the order of wetting rates of the three systems in the previous chapter. When the liquid moves on the solid surface, the liquid molecules desorb and tend to move forward, while the solid adsorbs and tends to fix the liquid molecules [41], which is the main reason for liquid wetting. Thus, when the bio-oil droplets move on the bitumen surface, the large $E_{bind}$ change indicates that more energy is absorbed to overcome the pinning effect of the bitumen surface on the droplets, and the droplets spread out more widely, which results in the wetting difference of the three bio-oils. How do microscopic interactions affect $E_{bind}$ and motion? We will discuss this in detail in the following sections.

#### 2.5.3. The Interactions between Bio-Oils Droplets and Bitumen Surface

**Figure 15:**
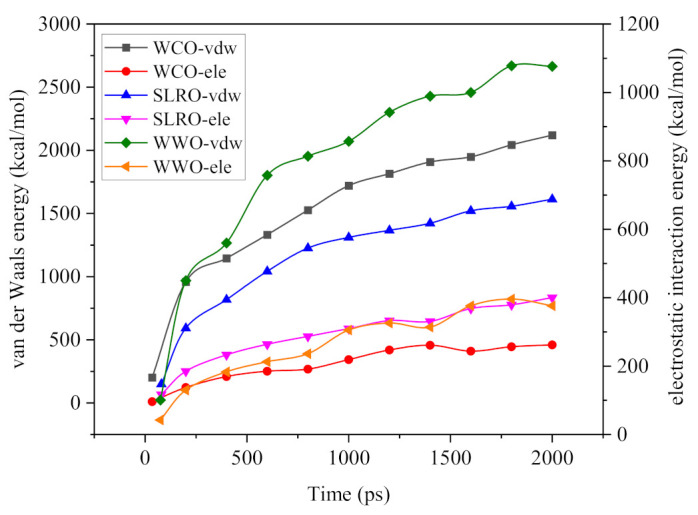
The E_vdw_ and E_ele_ between bio-oil droplets and aged bitumen surface.

As we all know, nonbond interactions at the micro level mainly include van der Waals interactions (E_vdw_) and electrostatic interactions (E_ele_). In this paper, the nonbond interaction between bio-oils droplets and the surface of aged bitumen is calculated to reveal the micro wetting mechanism, and the results are shown in Figure 15. It can be seen from Figure 15 that during the wetting process, the van der Waals energy and electrostatic energy gradually increase with the extension of the simulation time, and at the end of the simulation, the energy gradually tends to stabilize. This is because the bio-oil droplets gradually expand on the surface of aged bitumen, and the contact area with the surface gradually increases. Compared with the change in electrostatic energy, the change in van der Waals energy in the wetting process is more significant, indicating that Evdw has a stronger role in driving the wetting of droplets. However, a larger Evdw does not mean a stronger wetting ability. As shown in Figure 15, the E_vdw_ of the three bio-oils is WWO > WCO > SLRO, but this is not consistent with the above wetting ability law. The main reasons will be explained later.

#### 2.5.4. Influence of Hydrogen Bond on Wetting Ability of Bio-Oils

**Figure 16:**
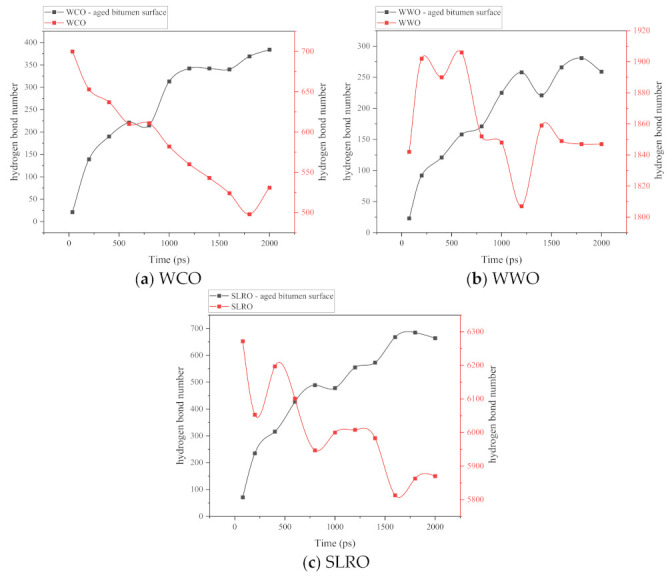
The number of hydrogen bonds in bio-oil droplets and between the droplets and aged bitumen surface.

During the wetting process of rejuvenators, the role of hydrogen bonds cannot be ignored. This mainly includes the hydrogen bond formed between the droplets and the surface of aged bitumen and the internal hydrogen bond of the droplets. The interfacial hydrogen bond can enhance the interaction between the rejuvenators and the bitumen surface, and the internal hydrogen bond of droplet determines its movement ability. In other words, the interfacial hydrogen bond is the driving force of wetting, while the hydrogen bond inside the droplet is the resistance; wetting is a process that destroys the internal hydrogen bonds of droplets. Therefore, in this work, the number of hydrogen bonds in the bio-oils droplets and the hydrogen bonds between the droplet and the aged bitumen surface are calculated during the wetting process, and the results are shown in Figure 16. From Figure 16, in general, with the progress of wetting, the number of hydrogen bonds between bio-oils and aged bitumen increases, while the number of hydrogen bonds within the three droplets decreases. However, there are some differences in the number of hydrogen bonds in the three wetting systems. First of all, for the interface, the number of hydrogen bonds eventually tends to stabilize, but the time to reach stability varies. The fastest is WCO, which indicates that the wetting time of WCO is the shortest. For droplets, the number of hydrogen bonds of the three bio-oils increases at the end of wetting, which is caused by the aggregation of molecules caused by droplet shrinking. Secondly, in addition to the monotonic decline in the number of hydrogen bonds in WCO, the number of hydrogen bonds in WWO and SLRO is in a fluctuating decline state, indicating that when WCO molecules are attracted by aged bitumen molecules, they are more difficult to keep spherical and easier to spread, so the number of hydrogen bonds hardly fluctuates. For WWO and SLRO, under the dual influence of the internal intermolecular attraction of liquid droplets and the attraction of bitumen molecules, they need to overcome the internal intermolecular attraction when wetting occurs. In order to keep spherical, they also need to overcome the attraction of bitumen molecules. This is the main reason why the number of hydrogen bonds will continue to fluctuate. Further, the number of hydrogen bonds in the three bio-oils also determines the strength of cohesion, which is consistent with the previous analysis of this paper. The more hydrogen bonds there are, the stronger the cohesion, and the less likely it is to wet. This also explains why WCO wets faster. In addition, comparing WWO and SLRO, at the beginning of wetting, the number of hydrogen bonds in WWO increases first and then decreases rapidly, which explains why the wetting angle in the early stage of WWO is larger than that in SLRO. On the other hand, it can be seen from Figure 16 that the number of hydrogen bonds in WWO and SLRO is absolutely superior to the number of interface hydrogen bonds, especially in SLRO. Compared with the analysis of van der Waals energy and electrostatic energy in the previous part, it is not difficult to find that the hydrogen bonds in the droplet are the main reason for the difference in wetting angle, and also the main reason for the poor wetting ability of WWO and SLRO.

### 2.6. Permeability Analysis of Bio-Oils

In this paper, the permeability of bio-oils is analyzed by testing the crossover modulus, which has been proved to be related to the dispersion degree of asphaltene and is an evaluation index for quantifying the polydispersity of asphaltene [42,43,44]. Our previous research found that both WWO and WCO can increase the crossover modulus, while SLRO can reduce the crossover modulus [45]. Therefore, comparing the crossover modulus of different PFLs with that of the original aged bitumen can determine the permeation position of the bio-oil in the aged bitumen.

#### 2.6.1. Effect of Permeation Time on PFL Crossover Modulus

**Figure 17:**
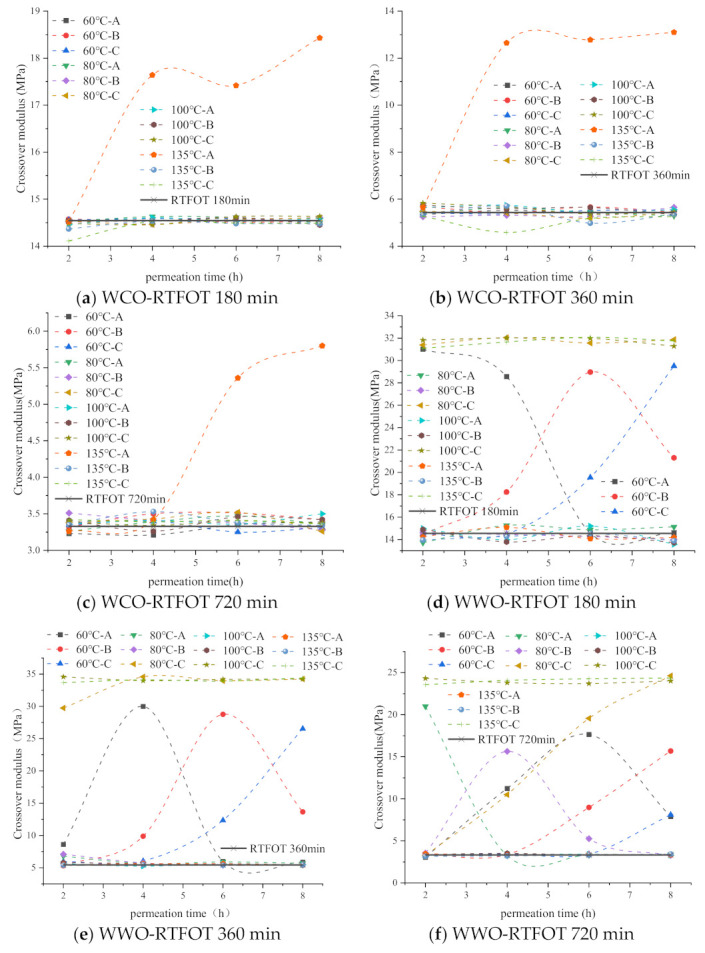

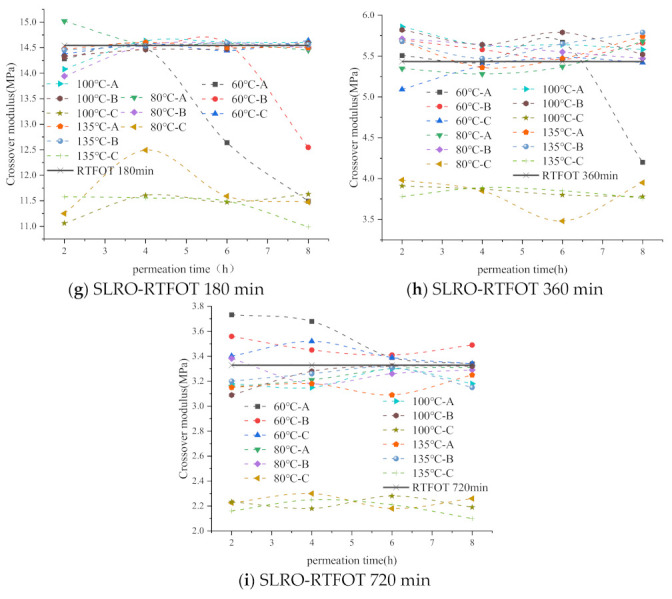
The crossover modulus of PFL varies with temperature and time.

In this paper, 60 °C, 80 °C, 100 °C and 135 °C are selected for the permeation test, and the changes in the crossover modulus of PFL with the penetration time are measured. The experimental results are shown in Figure 17. It can be seen from Figure 17 that for WCO-bitumen system, except for 135 °C, the crossover modulus of each PFL has not changed significantly with time. The crossover modulus of A, B and C is basically the same as that of aged bitumen, indicating that WCO has not permeated aged bitumen, as this requires a longer permeation time. At 135 °C, when the permeation time is 4–8 h, the crossover modulus of A in RTFOT 180 min and RTFOT 360 min is significantly higher than B and C, whose crossover modulus is still in the aged bitumen stage. For RTFOT 720 min, the crossover modulus of A is higher than B and C after 6–8 h permeation, and WCO permeates into layer A. The above results show that the extension of time is conducive to the permeation of WCO, but the permeability of WCO is poor, and it will take a longer time to permeate the bitumen substrate.

For the WWO-bitumen system, when the temperature is 60 °C, within 2 h, the crossover modulus of A in RTFOT 180 min increases significantly, the crossover modulus of A in RTFOT 360 min increases slightly, and the crossover modulus of A in RTFOT 720 min does not change, indicating that WWO can fully permeate A of RTFOT 180 min, and partially permeate A of RTFOT 360 min. Thereafter, with the extension of time, the crossover modulus of Part A in the aged bitumen gradually decreases, while Parts B and C gradually increase, and WWO gradually permeates B and C. When the temperature is 80 °C, for RTFOT 180 min and RTFOT 360 min, the crossover modulus of layer C reaches the maximum within 2 h, indicating that WWO has permeated layer C. For RTFOT 720 min, the crossover modulus of layer A is the largest within 2 h, and it increases gradually after 4–8 h, and WWO gradually permeates into layers B and C.

For the SLRO-bitumen system, at 60 °C, with the extension of time, the crossover modulus of any layer of RTFOT 720 min basically remains unchanged, indicating that SLRO cannot permeate RTFOT 720 min within 8 h, which requires a longer time. For RTFOT 180 min, the crossover modulus of A, B and C does not change within 2–4 h. When the time is 6 h, the crossover modulus of A is lower than that of the aged bitumen, indicating that SLRO can start to permeate RTFOT 180 min only after 6 h. For RTFOT 360 min, this time can only be started after 8 h. After 80 °C, when the permeation time is 2 h, the crossover modulus of C in the three aged bitumen is lower than A and B, indicating that SLRO can permeate to C within 2 h.

In conclusion, among the three kinds of bio-oil, WWO needs the shortest time to permeate bitumen with different aging degrees, followed by SLRO, and WCO has the worst permeability.

#### 2.6.2. Effect of Permeation Temperature on PFL Crossover Modulus

It can be seen from Figure 17 that with the increase in temperature, the permeability of the three kinds of bio-oil in the aged bitumen increases, and the permeability at 60 °C for a long time is equivalent to that above 80 °C for a short time. By comparing the crossover modulus within 2 h, WWO and SLRO can significantly affect the crossover modulus of layer C, indicating that the two kinds of bio-oil can permeate layer C at 80 °C, and the crossover modulus of each layer almost does not change after 80 °C. However, for WCO, during 4–8 h, it only has a significant effect on the crossover modulus of A at 135 °C. In a word, the increase in temperature is beneficial to the permeability of bio-oil. The permeability of WCO is far lower than that of WWO and SLRO, and the permeability of WWO is the best.

#### 2.6.3. Effect of Aging Degree on PFL Crossover Modulus

It can be seen from Figure 17 that with the severity of bitumen aging, the permeability of bio-oils in aged bitumen decreases. Take the permeation of WWO in aged bitumen at 60 °C, for example. After 2 h, the crossover modulus of A in RTFOT 180 min reaches the maximum, and the crossover modulus of A in RTFOT 360 min also increases to some extent, but the crossover modulus of any section of RTFOT 720 min is basically in the initial aged bitumen state. This shows that WWO can completely permeate layer A of RTFOT 180 min and partially permeate layer A of RTFOT 360 min, but cannot permeate RTFOT 720 min. After 4–8 h permeation, the crossover modulus of B and C in RTFOT 180 min and RTFOT 360 min increases to different extents. For RTFOT 720 min, only the crossover modulus of A and B increase after 6 h, but the crossover modulus of C does not change. The crossover modulus of C does not increase until 8 h, indicating that WWO is more likely to permeate RTFOT 180 min and RTFOT 360 min, and it will find it more difficult to permeate RTFOT 720 min. Based on the above analysis, the aging degree will weaken the permeability of rejuvenators. The more serious the aging, the more difficult the permeation and the longer the time. This is because the more serious the aging, the greater the presence of polar components such as asphaltene, the stronger the intermolecular interaction, the greater the cohesion, the harder the asphalt and the greater the resistance of the rejuvenators to permeate the aged bitumen.

#### 2.6.4. Effect of PFL Layer on Crossover Modulus

**Figure 18:**
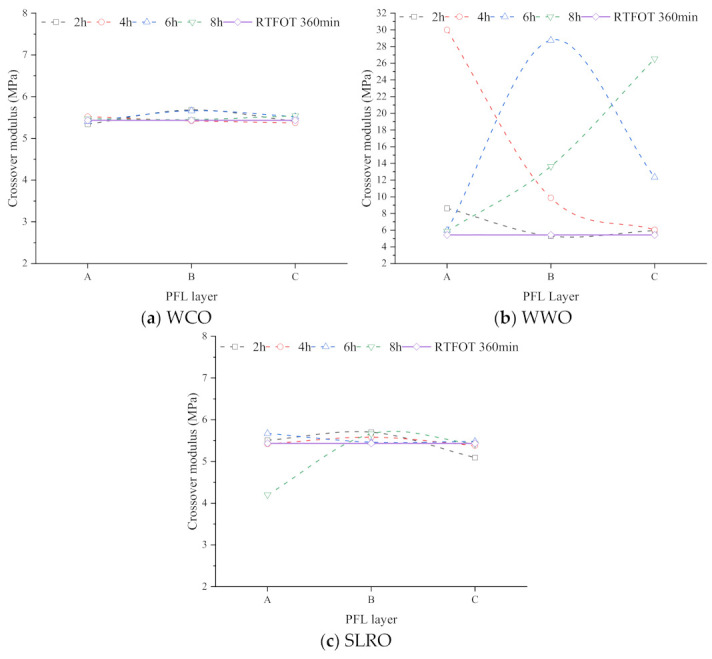
Effect of PFL layer on crossover modulus.

During aged bitumen regeneration, the permeability of the rejuvenator in the aged bitumen also depends on the bitumen film thickness. With the increase in the bitumen film thickness, the permeation process of the rejuvenator gradually deepens, so the crossover modulus of different layers will be different. Figure 18 shows the crossover modulus of different layers in RTFOT 360 min at 60 °C. For WCO-RTFOT 360 min, the crossover modulus hardly changes with the depth of the layer, indicating that WCO does not permeate any layer. For WWO-RTFOT 360 min, when the permeation time is less than or equal to 4 h, the crossover modulus decreases with the deepening of the layer, indicating that the thicker the bitumen layer is, the harder it is for the bio-oil to reach the layer. Compared with B and C, the crossover modulus of layer A greatly exceeds that of the two layers, indicating that the bio-oil mainly exists in layer A. However, as time goes on, the crossover modulus of B and C increases and the crossover modulus of layer A decreases after 6–8 h, indicating that bio-oil permeates into B and C. After 8 h, the crossover modulus of C reaches maximum, and that of layer B decreases significantly, indicating that the bio-oil mainly exists in layer C. It can be seen from the above analysis that when the thickness of the bitumen layer is small, the bio-oil easily permeates the bottom layer. When the thickness is greater, it takes a long time to permeate the bottom layer. In the actual recycling project, the thickness of bitumen film is not uniform, so it is important to select a good permeable rejuvenator. Among the three bio-oils in this paper, WWO has excellent permeability, which can be used as a choice of regenerant.

Based on the analysis of Section 2.6.1, Section 2.6.2, Section 2.6.3, Section 2.6.4, it can be seen that for WCO, it permeates at 135 ℃ for 4–8 h into the A layer of RTFOT 180 min and RTFOT 360 min and permeates into the A layer of RTFOT 720 min in 6–8 h. For WWO, it permeates at 60 °C for 6–8 h into the C layer of RTFOT 180 min and RTFOT 360 min and permeates into the C layer of RTFOT 720 min in 8 h. When the temperature is greater than 80 °C, it permeates into the C layer of RTFOT 180 min and RTFOT 360 min in 2 h and permeates into the C layer of RTFOT 720 min in 4 h. For SLRO, it permeates at 60 °C for 8 h into layer B and layer A of RTFOT 180 min and RTFOT 360 min, respectively; when the temperature is greater than 80 °C, it permeates into the layer C of RTFOT 180 min, RTFOT 360 min and RTFOT 720 min within 2 h. In the actual regeneration project, it is assumed that the thickness of aged bitumen film coated on the RAP surface is generally 20–30 μm [5]. However, this paper adopted a 6 cm bitumen layer, only WWO and SLRO permeated the bottom layer within the specified temperature and time, while WCO struggled to penetrate the bitumen bottom layer. Therefore, based on the actual thickness (20–30 μm) of the aged bitumen in the actual regeneration project, the approximate mixing time for WWO and SLRO to permeate the aged bitumen film coated on the RAP surface is as follows: For WWO, the mixing time of WWO and RAP (RTFOT 180 min, RTFOT 360 min and RTFOT 720 min) is about 9.6–14.4 s at 60 °C. When the temperature is higher than 80 °C, the mixing time of WWO and RAP (RTFOT 180 min, RTFOT 360 min) is about 2.4–3.6 s. The mixing time with RAP (RTFOT 720 min) is about 4.8–7.2 s. For SLRO, the mixing time of SLRO and RAP (RTFOT 180 min, RTFOT 360 min and RTFOT 720 min) is about 2.4–3.6 s when the temperature is higher than 80 °C.

#### 2.6.5. Micromorphology Analysis of PFL

**Figure 19:**
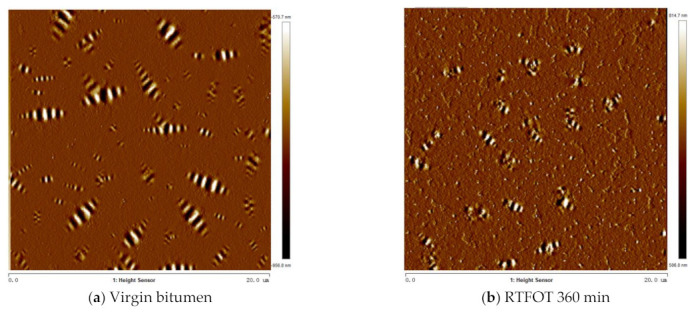

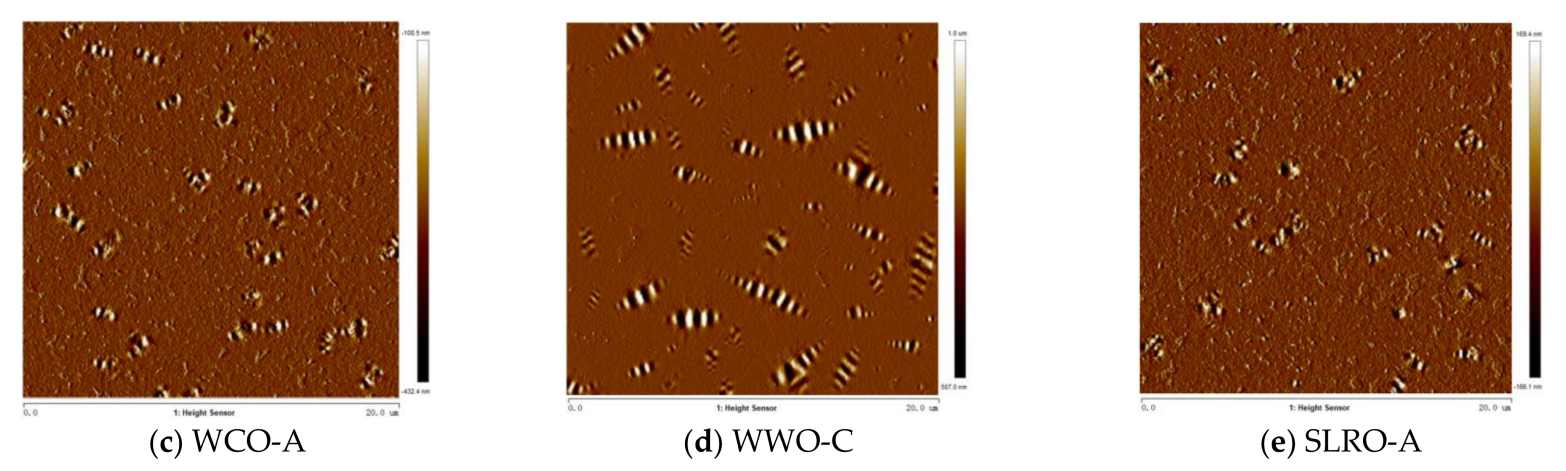
Effect of bio-oil on micromorphology of PFL.

In order to evaluate the influence of three kinds of bio-oils on the microscopic morphology of PFL during the permeation process, this section selects the PFL after the permeation of three kinds of bio-oils in RTFOT360 min at 60 °C after 8 h as an example. Figure 17 shows the micromorphology of PFL before and after bio-oil permeates. It can be seen from Section 2.6.1 that after 8 h, WWO and SLRO significantly affect the crossover modulus of layer C and layer A, respectively, while WCO has no effect on the crossover modulus of any layer. Therefore, this section only uses A and C as examples. It can be seen from Figure 19 that after aging for 360 min, the micromorphology of bitumen changes from a uniformly distributed bee structure to a more concentrated and incomplete bee structure. Relevant research shows that the formation mechanism of bee structure is due to the formation of highly polar asphaltenes associated with polymer microcrystalline wax, part of the resin, and a small amount of oil dispersed in the continuous phase [46,47]. Other researchers believe that during the cooling process of bitumen, it is influenced by the strong polarity and high molecular weight of asphaltene to precipitate and form a bee structure [48]. Regardless of the viewpoint, it is indicated that asphaltene is one of the main factors promoting the formation of bee structure [47,49]. After bitumen aging, the content of polar molecules such as asphaltene increases [24], while the content of light components such as aromatic components decreases [23], and macromolecular substances will reduce the molecular diffusion ability, thus inhibiting the bee structure [50]. Therefore, the bee structure in aged bitumen is incomplete. In Figure 19c,e, the bee appearance structure is still incomplete, indicating that WCO and SLRO do not play a positive role in regeneration, and the movement of macromolecules in bitumen is still limited. The number of bee structures after adding SLRO is less than that of RTFOT 360 min, indicating that SLRO promotes the inhibition of macromolecular activity. However, the appearance of PFL with WWO is basically restored to the virgin bitumen state, which indicates that WWO activates the macromolecular surface activity, promotes the mobility between the chain segment and macromolecular structure and makes it evenly dispersed in the bitumen, leading to the complete appearance of the bee structure.

#### 2.6.6. Molecular Morphology Analysis of Bio-Oil during Permeation

**Figure 20:**
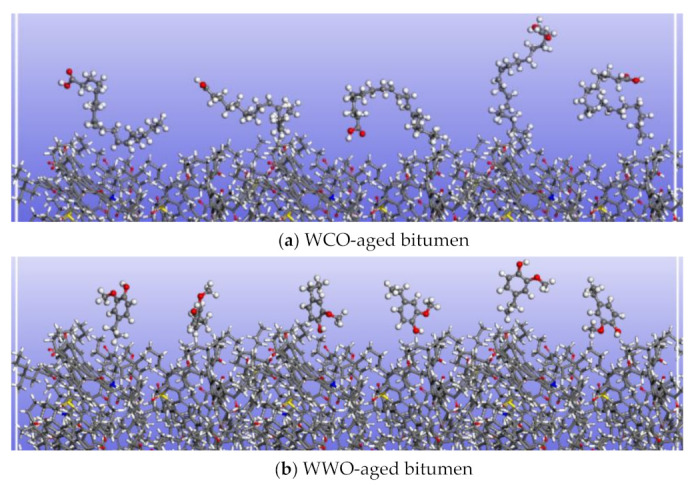

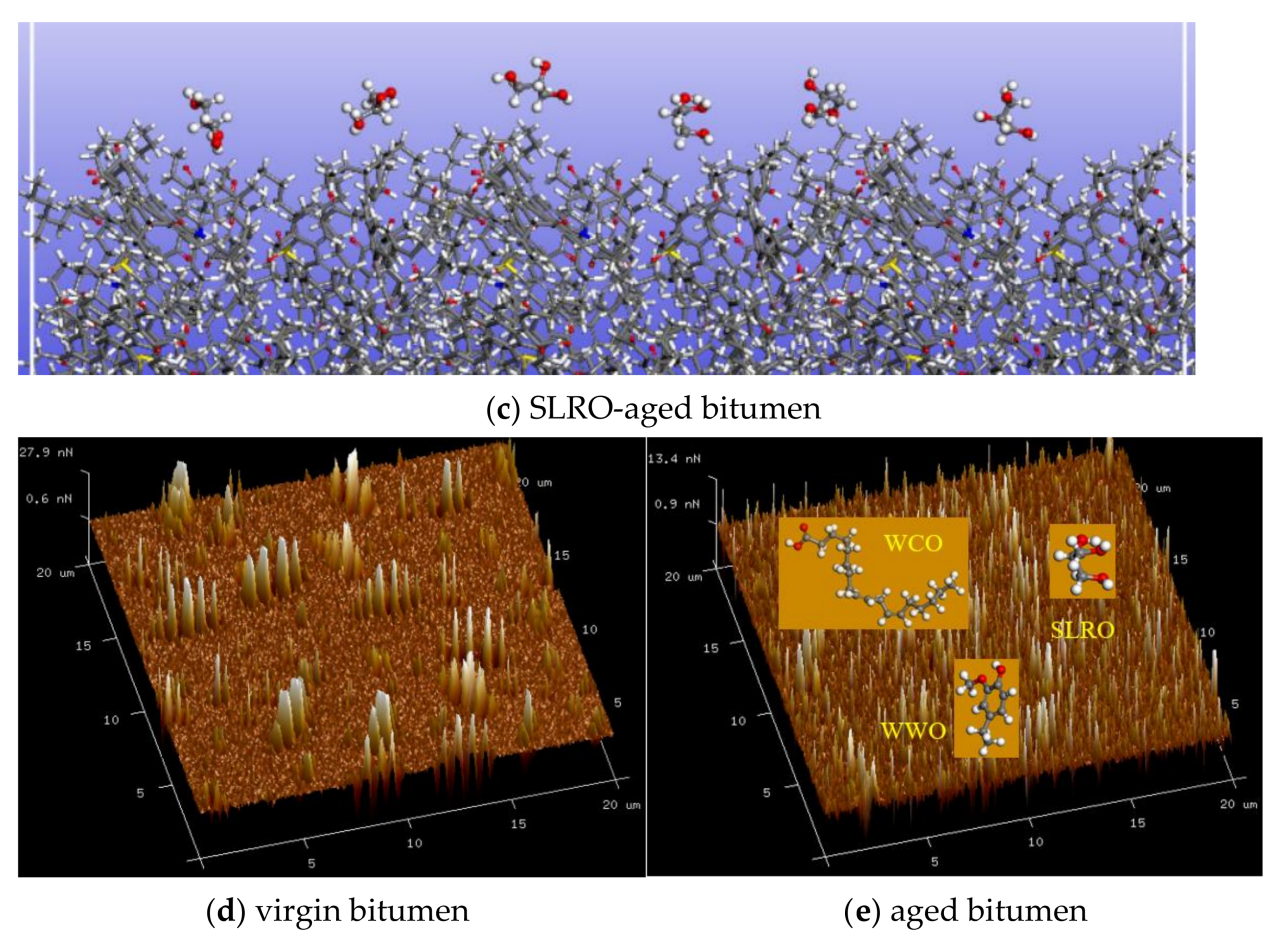
Molecular morphology of bio-oil during permeation.

Figure 20 shows the morphology of the interface between bio-oil molecules and aged bitumen during the permeation process. As can be seen from Figure 20, the molecular morphologies of the three bio-oil molecules present different states when they are in contact with the bitumen surface during the infiltration process. After coming into contact with the bitumen surface, most of the WCO molecules are similarly parallel to the bitumen surface, and only a few molecules are perpendicular to the bitumen surface. WWO molecules are basically perpendicular to the bitumen surface, while SLRO molecules have different forms. Molecular morphology determines the degree of permeation. Due to the increase in polar functional groups after bitumen aging, the intermolecular attraction is enhanced and the molecular distribution is denser, as shown in Figure 20d,e. This makes it more difficult for bio-oil molecules to interpenetrate bitumen molecules. From the morphology of bio-oil, WWO molecules perpendicular to the bitumen surface more easily penetrate the bitumen. Our previous research shows that WWO shows an intercalation effect, and there is a repulsive force between WWO and asphaltene molecules, which makes WWO molecules easier to penetrate. However, most WCO molecules are parallel to the bitumen surface, making it difficult to insert them into the bitumen molecules, so the permeability of WCO is poor. Although SLRO has different morphologies, its molecular chain is very short and easy to insert into the pores between bitumen molecules, so SLRO can easily penetrate bitumen.

## 3. Materials and Methods

### 3.1. Preparation of Aged Bitumen

In this paper, No. 90 virgin bitumen is selected. The basic performance indexes of bitumen are shown in Table 2. Aged bitumen samples were prepared via rotary film oven test (RTFOT). Related research shows that RTFOT aging time extension has a high correlation with the PAV aging test. The long-term aging effect of PAV-simulated asphalt pavement is equivalent to the effect of RTFOT aging 270 min; the RTFOT aging for 180 min is equivalent to the aging degree of actual operation 2–3 years after pavement construction and the RTFOT aging for 360 min is basically equivalent to the aging degree of the actual pavement used for 6 years; RTFOT+PAV aging is roughly equivalent to the actual pavement aging effect of 4–5 years [51]. Therefore, five kinds of aged bitumen (RTFOT 85 min, RTFOT 180 min, RTFOT 360 min and RTFOT 720 min) were prepared to simulate bitumen with different aging degrees.

### 3.2. Molecular Models

**Figure 21:**
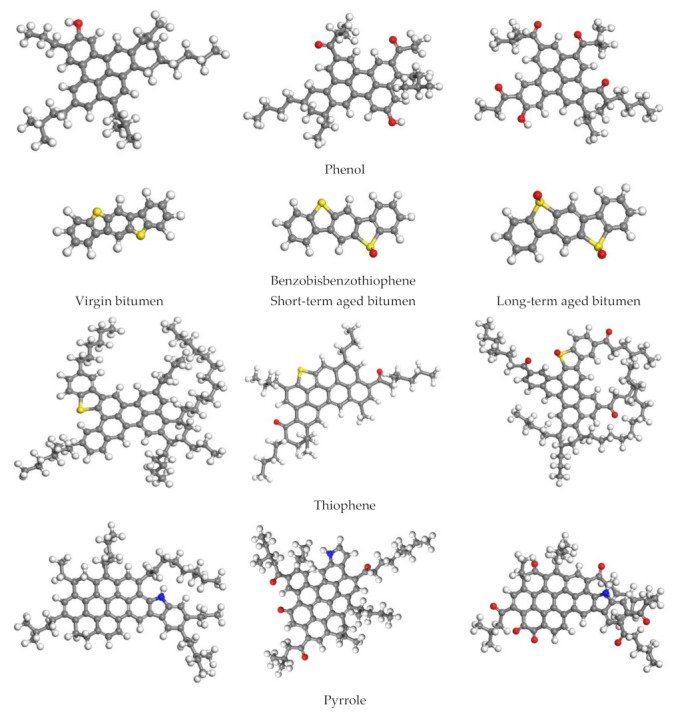

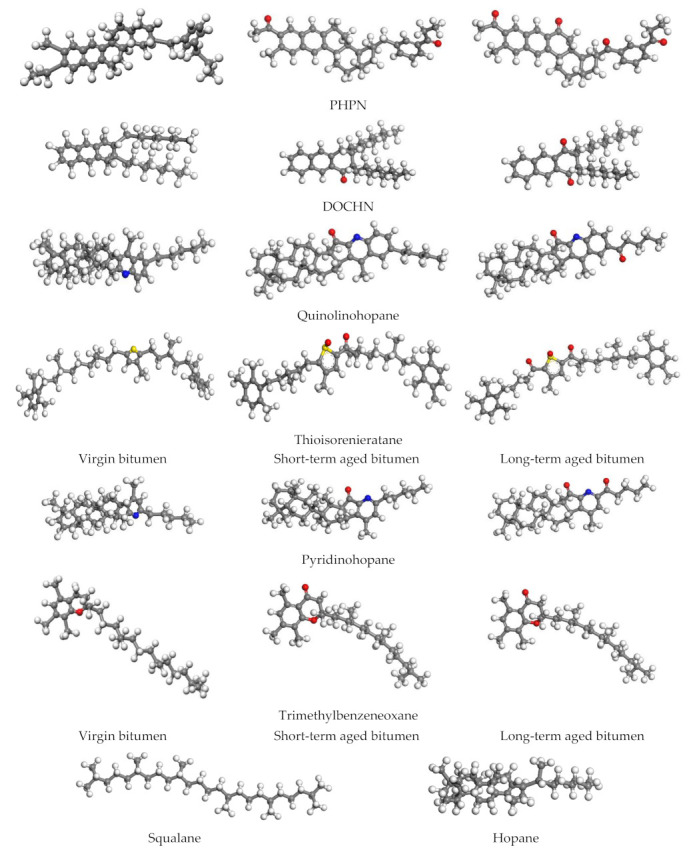
Molecular structures of 12-component bitumen model with different aging levels. (Carbon atoms are grey, hydrogen atoms are white, oxygen atoms are red, sulphur atoms are yellow, and blue is nitrogen).

Bitumen is a complex organic mixture of hydrocarbons and nonhydrocarbons, with relatively large molecular weight. In order to study the chemical composition of bitumen, people have carried out a lot of research work, including nuclear magnetic resonance (NMR), small-angle neutron scattering (SANS) and small-angle X-ray scattering (SAXS) [52]. However, due to the complex chemical composition and diverse sources of bitumen, the specific molecular formula of bitumen is still uncertain and divergent [53]. Therefore, in the past MD simulation, some representative molecular structures were selected to represent the complex bitumen composition. In this study, the 12-component molecular model proposed by Li and Greenfield is used to represent the virgin bitumen molecules [54]. For aged bitumen, ketones and sulfoxides have been used in many studies to replace the sensitive functional groups in the virgin bitumen. Considering the oxidation degree of bitumen, it is also recommended to use different amounts of ketone and sulfoxide functional groups [15,55]. Based on these studies, the 12-component model of long-term aged bitumen is determined in this paper. In the process of establishing the short-term aged bitumen model, Qu et al. added some oxygen atoms to the virgin bitumen model to indicate the increase in ketone and sulfoxide functional groups according to the FITR results and the long-term aged bitumen model, and thus established the short-term aged bitumen model [37], and this model has been applied and verified in the research of Sun et al. [13]. The molecular structures of 12-component of virgin bitumen, short-term aged bitumen and long-term aged bitumen are shown in Figure 21. In addition, the asphaltene structure proposed by Martín et al. [56] is used to replace the molecular structure proposed by Li and Greenfield. This is because the asphaltene molecular structure proposed by Li enables the bitumen system to have higher energy. In the actual chemical reaction, it is difficult to keep stable for a long time. Table 3 and Table 4 list detailed bitumen molecular information.

**Figure 22:**
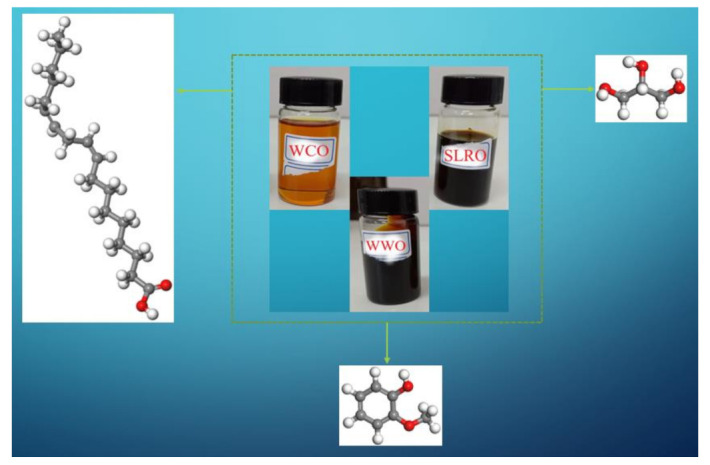
Molecular structure of bio-oils. (Carbon atoms are grey, hydrogen atoms are white, and oxygen atoms are red).

Similar to bitumen, the chemical composition of biological extract is also extremely complex. Even though most of the chemical components can be extracted by GC-MS or NMR tests, it is difficult to determine the molecular structure of the substance due to the complexity of the components. Therefore, in most studies, the component or main component with the highest measured content in the test represents the substance [23,24,27,57,58]. In our previous studies, the main components of three bio-oils (WCO, WWO and SLRO) were measured by GC-MS test [45], and the molecular model of bio-oil was constructed by selecting substances with higher content through analysis. The molecular structures are shown in Figure 22.

### 3.3. Wetting Simulation

Bitumen wettability is the tendency of bio-oils droplets to spread on or adhere to bitumen surfaces. It reflects the intermolecular interaction when two different materials are knitted together. Wettability can be measured by the contact angle after thermodynamic equilibrium, which is determined by the balance between adhesion (between bio-oil droplets and bitumen surface) and cohesion (inside bio-oil droplets). The contact angle is one of the main indexes used to evaluate the adhesion between two phases [59,60,61]. A contact angle less than 90° means that the droplet can wet the bitumen surface. When the contact angle is greater than 90°, the bitumen surface is difficult for the droplet to wet. The greater the contact angle, the more difficult the bitumen surface is to be wetted.

MD simulation was used to investigate the wetting behavior of bio-oils on different aged bitumen surfaces in our research. The virgin bitumen and different aged bitumen models are built through Materials Studio, mainly in the following steps: (1) We randomly allocate molecules in the periodic lattice through the Amorphous Cell module, and the initial density is set to 0.1 g/cm^3^. (2) Geometric optimization is conducted to minimize system energy. (3) In the NVT ensemble, we run the system for 50 ps, then set the temperature at 500 K, with a step size of 0.1 fs. (4) In the NVT ensemble, we continue to run the system for 300 ps, with the step size set to 1 fs and the temperature set to 500 K . The purpose of this step and the previous step is to accelerate the molecular motion and promote the random combination of molecules, so as to make the model more reasonable. (5) In the NPT ensemble, we set the temperature to 500 K and the step size to 1 fs at 1 standard atmosphere to make the system run for 500 ps. (6) In the NPT ensemble, the system runs for 2 ns to achieve density balance. (7) In the NVT ensemble, the bitumen system continues to run for 2 ns and further relaxes the molecules to make the system energy more stable. When the bitumen system is stable, the lattice parameters are increased by 5 and 4 times, respectively, in the X and Y directions through supercell, and the structure is optimized to minimize the system energy. Then, the expanded system is run for 2 ns in the NVT ensemble to make the system reach equilibrium.

**Figure 23:**
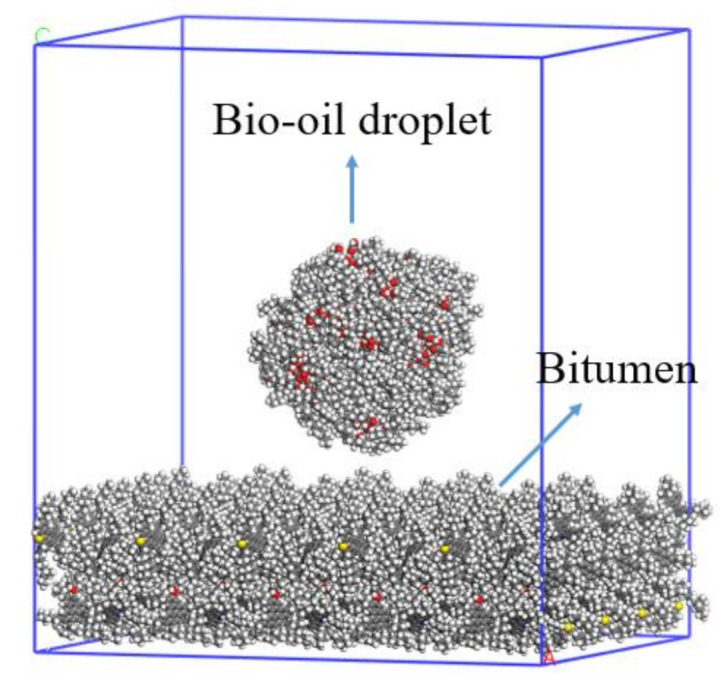
Bio-oil–bitumen interface wetting model.

Additionally, three bio-oil nano-droplets with 28 Å radius were constructed in a spherical simulation cell with the Amorphous Cell tool. A 200 ps MD simulation with the time step of 1 fs at temperature 298 K under the NVT ensemble was carried out to obtain the stable bio-oils nano-droplets. After thermodynamic equilibrium, the bitumen wetting model was constructed by placing the bio-oils nano-droplets over the center of bitumen surfaces with a distance of about 5 Å between the bio-oils nano-droplets and the bitumen surfaces. At the same time, in order to prevent the influence of periodic structure, a 50 Å vacuum layer was set in the Z direction. Figure 23 takes the WCO-virgin bitumen surface as an example to show the bitumen wetting model. After the bitumen wetting models were established, each model was optimized, and followed by equilibrium for 2 ns under the constant volume, constant temperature (298 K) NVT ensemble with the Nosé-Hoover thermostat. The Particle–Particle–Particle Mesh (PPPM) algorithm was employed to calculate the long-range interaction and electrostatic energy [36].

### 3.4. Contact Angle Test

**Figure 24:**
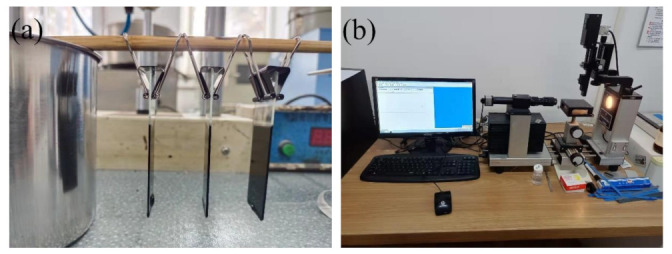
Contact angle test: (**a**) Bitumen film sample; (**b**) OCA 20 video optical contact angle measuring instrument.

The sample preparation procedure is as follows: firstly, the virgin bitumen and aged bitumen are heated to the flowing state, and then some 25.4 mm × 76.2 mm × 1 mm slides preheated in an oven at 135 °C are taken out. Secondly, the slide is inserted into the flowing bitumen. Finally, the slide is clamped to allow the bitumen to flow naturally downward along the slide, and then put into an oven at 70 °C for 30 min to obtain a bitumen film sample with smooth surface (Figure 24a). After the sample is cooled, the sample is placed on the OCA 20 video optical contact angle measuring instrument (Figure 24b) using the hanging drop method for testing.

### 3.5. Surface Wetting Theory

**Figure 25:**
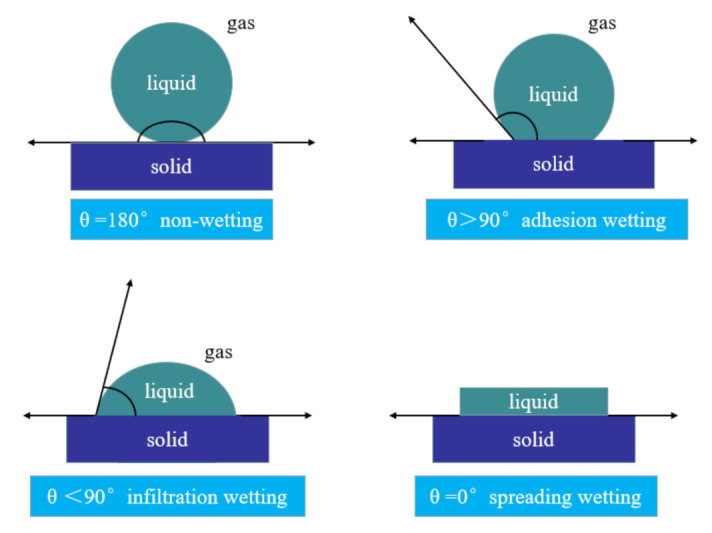
Droplet wetting process.

Surface wettability is an inherent and important characteristic of a solid surface. Wetting generally refers to the process of one fluid replacing another from the solid surface. The wetting process is a thermodynamic process, and the change in surface free energy determines whether the wetting process can proceed spontaneously and reflects the resistance in the wetting process [62,63]. The solid/gas interface is formed by the contact between solid and gas. A drop of liquid drops on the solid surface, and the solid/liquid interface formed after the solid and liquid contact replaces part of the solid/gas interface, which is a wetting process. According to the interface behavior characteristics of the wetting process, the wetting types can be divided into adhesion wetting, infiltration and spreading wetting [6,62,63], as shown in Figure 25.

#### 3.5.1. Adhesion Wetting

The process of liquid replacing the gas on the solid surface but not spreading completely is called adhesion wetting. In this process, the surface free energy of the whole system changes as follows:

(2) $$\Delta G=\gamma_{SL}-\gamma_{SV}-\gamma_{LV}=-W_{a}$$

where $\gamma_{SL}$ is solid–liquid interfacial tension; $\gamma_{SV}$ is the interfacial tension between solid and gas, which is the free energy of solid surface; $\gamma_{LV}$ is gas–liquid interfacial tension, that is, the surface tension of the liquid itself; $W_{a}$ the adhesion work, and the value of the adhesion work indicates the firmness of the solid–liquid two-phase spreading and bonding [64].

#### 3.5.2. Infiltration Process

The immersion of solid into liquid is called infiltration. In this process, the solid–gas interface is replaced by the solid–liquid interface, and the gas–liquid interface does not change, so the system free energy changes as follows:

(3) $$\Delta G=\gamma_{SL}-\gamma_{SV}=-W_{i}$$

where $W_{i}$ is infiltration work. Unlike the adhesion wetting process, infiltration can not occur between all liquids and solids. The infiltration process can proceed spontaneously only when the solid–liquid interfacial tension is lower than the free energy of the solid surface.

#### 3.5.3. Spreading Wetting

When the temperature and pressure are constant and there is no external force, if the droplet can develop on the solid surface to form a liquid film, it is considered that the liquid can spread and wet on the solid surface. For spreading wetting, spreading coefficient S is commonly used to represent the change of system free energy, and the change in system surface free energy is as follows:

(4) $$\Delta G=\gamma_{SL}+\gamma_{LV}-\gamma_{SV}=-S=W_{c}-W_{a}$$

where $W_{c}$ is the cohesion work of the liquid.

If S ≥ 0, then ΔG ≤ 0, and the liquid can automatically spread on the solid surface. When the cohesion work of the liquid is smaller than the adhesion work between the liquid and the solid, the liquid can spontaneously unfold on the solid surface.

The droplet L is placed on an ideal plane S. If one phase is gas V, the contact angle is the angle at which the gas–liquid interface intersects the solid–liquid interface through the liquid. According to Young, the balance of interfacial tension at the solid–liquid-gas interface leads to the formation of contact angles. When the three forces are in equilibrium, there should be the following relationship:

(5) $$\gamma_{SV}=\gamma_{SL}+\gamma_{LV}cos\left(\theta \right)$$

Equation (5) is Young’s equation, where $\gamma_{SV}$ and $\gamma_{LV}$ are the surface tension (or surface free energy) of a solid and a liquid under saturated vapor pressure. In general, the surface free energy and the solid–liquid interfacial tension are difficult to measure in experiments, while the contact angle introduced by Young’s equation is easy to obtain. By combining Equation (5) with Equations (2)–(4), the relationship between the contact angle and adhesion work, infiltration work and spreading coefficient S can be obtained, as shown in Equations (6)–(8).

(6) $$W_{a}=\gamma_{LV}\left(cos\left(\theta \right)+1\right)$$

(7) $$W_{i}=\gamma_{LV}cos\left(\theta \right)$$

(8) $$S=\gamma_{LV}\left(cos\left(\theta \right)-1\right)$$

According to Equations (6)–(8), the wettability of a liquid on a solid surface can be preliminarily judged by the contact angle of the liquid on the solid surface. The values of $W_{a},\;\;W_{i}$, S are determined by the liquid surface tension and the contact angle θ. Once the liquid is determined, its surface tension, as a characteristic of the liquid itself, remains a certain value under constant conditions, and the change in free energy in the wetting process is completely affected by the contact angle θ. Therefore, the contact angle θ can also be used as the criterion for judging the wettability of liquid on solid surface.

#### 3.5.4. Wetting Velocity and Wetting Time

When the liquid wets the solid surface, the wettability can be evaluated by the wetting velocity and time of the liquid on the solid surface. If the pores on the solid surface are regarded as capillaries, the velocity of liquid flowing through the capillaries and the required time can be calculated according to the surface tension, the viscosity of the liquid, the contact angle between the liquid and the solid and the surface morphology of the solid [65]. The calculation formula of wetting velocity and wetting time is as follows:

(9) $$W_{v}=\frac{dL}{dt}=\frac{\left(\gamma_{S}-\gamma_{SL}\right)R}{4\eta L}=\frac{R\gamma_{L}cos\left(\theta \right)}{4\eta L}$$

(10) $$W_{t}=\frac{2\eta L^{2}}{R\left(\gamma_{S}-\gamma_{SL}\right)}=\frac{2\eta L^{2}}{R\gamma_{L}cos\left(\theta \right)}$$

where $W_{v}$ is the wetting velocity; $W_{t}$ is the wetting time; θ is the contact angle of bio-oil droplets on bitumen surface at equilibrium; $\gamma_{L}$ is the surface tension of bio-oil; η is the viscosity of bio-oil; L is the capillary length of aged bitumen surface; R is the capillary radius of the aged bitumen surface. The surface structure of the aged bitumen prepared under the same conditions is basically the same. For the convenience of calculation, this paper assumes that R and L are both 1, then the wetting velocity is $W_{v}=\gamma_{L}cos\theta /4\eta$, and the wetting time per unit length is $W_{t}=2\eta /\gamma_{L}cos\theta$.

### 3.6. Bio-Oils Permeation Test

#### 3.6.1. Preparation of Permeation Fusion Layer (PFL)

**Figure 26:**
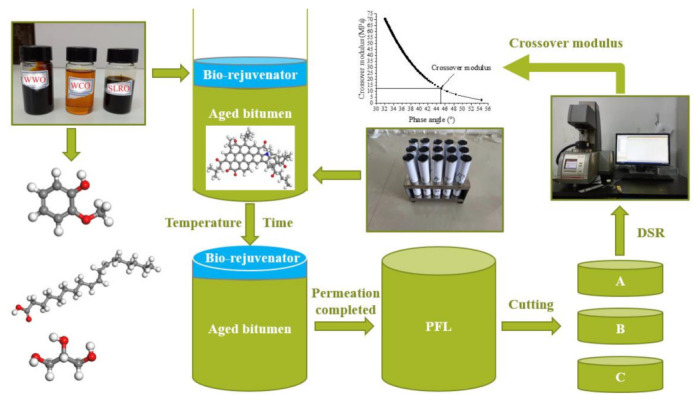
Preparation of PFL.

In order to explore the permeability of bio-oils in bitumen with different aging degrees, the PFL samples were obtained through permeation tests (Figure 26). The experimental temperatures were 60 °C, 80 °C, 100 °C and 135 °C, and each temperature lasted for 2 h, 4 h, 6 h and 8 h, respectively. When the experiment was completed, the bitumen was cut into three equal parts which were named A, B and C, respectively. Then, the permeability of the bio-oil in the aged bitumen was analyzed by testing the crossover modulus of different layers. Three kinds of aged bitumen, RTFOT 180 min, RTFOT 360 min and RTFOT 720 min, were selected. In the permeation test, the mass of aged bitumen and bio-oil was 30 g (about 6 cm) and 2.4 g, respectively.

#### 3.6.2. Dynamic Shear Rheometer Test

An Anton Paar Rheometer MCR 302 was used to measure the elastic behavior and viscous behavior of each sample following ASTM D7175. For this study, an 8 mm parallel plate spindle was used. Using the corresponding storage modulus (G′) and loss modulus (G″) results, the crossover modulus (at which G′ is equal to G″) were determined. The phase angle corresponding to the crossover modulus was 45°. Tests were conducted at 10 °C with frequencies of 0.1–100 rad/s and at a strain rate of 0.1%. The reason why 10 °C is selected as the test temperature is that for virgin bitumen, when the temperature is high, the phase angle will exceed 45°. When the temperature is low, the phase angle will be lower than 45°. These two conditions are not conducive to obtaining the crossover modulus. After many tests, 10 °C is the appropriate temperature; this has also been confirmed by many previous studies [23,66,67].

#### 3.6.3. Atomic Force Microscope (AFM) Test

**Figure 27:**
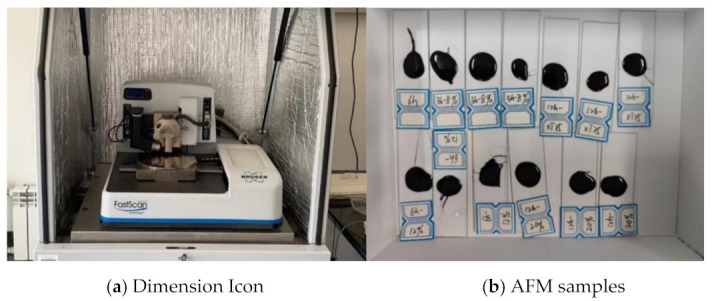
Dimension Icon-type atomic force microscope and PFLs samples.

The AFM test is used to observe the micro morphology of the PFLs to evaluate the permeability and regeneration effect of bio-oils. The instrument (Figure 27a) is the Dimension Icon-type atomic force microscope produced by Brooke Company. After the PFLs were obtained, a heated spoon was used to cut 5 g of the corresponding bitumen from PFLs and transfer it to the metal canister. The canister was covered and placed in a preheated oven set at 130 °C. After 5 min of heating with little oxidation, a thermometer was used to homogenize the liquid bitumen. Three droplets were coated on three cavities of a microscope slide for microscopic observation. The microscope slide was placed on a preheated plate of 120 °C for 5 min. This allowed the bitumen droplets to spread and form a thin, flat film (Figure 27b). After the slide was removed from the heating plate, it was placed in a crystallizing dish and covered with a metal lid. The samples used for microscopy were covered with metal lids (to prevent dust contamination) and stored at room temperature in a dark environment for 24 h. The resting time for microscopy tests were selected based on previous experiences and literature [68,69]. The scanning range was 20 μm × 20 μm.

## 4. Conclusions

The study comprehensively analyzed the wetting and permeability of three kinds of bio-oils, providing a theoretical basis for effectively selecting rejuvenators suitable for aged bitumen regeneration. The factors influencing the wetting and permeability of bio-oil are revealed in depth. The main conclusions are as follows:

(1) The degree of aging significantly affects the wettability of bio-oil on the bitumen surface. The more serious the aging, the more difficult the bio-oil is to spread on the bitumen surface, and the more unfavorable the regeneration of aged bitumen. In addition, high temperatures are more conducive to the wetting behavior of bio-oil than low temperatures.
(2) Large viscosities are not conducive to the wetting of bio-oil on the bitumen surface, while large surface tension is conducive to the spread of bio-oil. Therefore, when selecting rejuvenators, those with low viscosity and high surface tension are preferred. In this paper, because WCO has the lowest viscosity, and its surface tension is higher than that of WWO and SLRO, it is more suitable as a rejuvenator for aged bitumen.
(3) When the bio-oil droplet spreads on the aged bitumen surface, a precursor film forms preferentially. Then, a phenomenon called “bio-oil molecules spread on precursor film” is observed. The microscopic driving and resisting force for the motion of the bio-oil droplet on the aged bitumen surface are analyzed. The van der Waals energy plays the major role for the bio-oil droplet motion. Meanwhile, the hydrogen bond interactions affect the motion as well.
(4) The permeation of bio-oil in aged bitumen is affected by temperature, permeation time, bio-oil type, bitumen layer thickness and bitumen aging degree. A higher temperature or a longer time is conducive to the permeation of bio-oil in aged bitumen. A thicker bitumen layer requires a longer permeation time. The increase in aging degree will inhibit the permeability of bio-oil. In this paper, the permeability of WWO in aged bitumen is the strongest, while WCO is the weakest.
(5) When WWO and SLRO are applied to bitumen regeneration, the wetting ability is best when the mixing temperature is no lower than 408.15 K. Based on the actual thickness (20–30 µm) of the aged bitumen in the actual regeneration project, the approximate mixing time for WWO and SLRO to permeate the aged bitumen film coated on the RAP surface is as follows. For WWO, the mixing time of WWO and RAP (RTFOT 180 min, RTFOT 360 min and RTFOT 720 min) is about 9.6–14.4 s at 60 °C. When the temperature is higher than 80 °C, the mixing time of WWO and RAP (RTFOT 180 min, RTFOT 360 min) is about 2.4–3.6 s. The mixing time with RAP (RTFOT 720 min) is about 4.8–7.2 s. For SLRO, the mixing time of SLRO and RAP (RTFOT 180 min, RTFOT 360 min and RTFOT 720 min) is about 2.4–3.6 s when the temperature is higher than 80 °C.
(6) The AFM test confirms that the permeation effect of WWO in aged bitumen is superior to that of WCO and SLRO from the microscopic perspective. WWO can activate the macromolecular surface activity and promotes the mobility between the chain segment and macromolecular structure in aged bitumen, giving scope to a favorable regenerative effect.
(7) During the penetration process, WWO molecules are distributed perpendicular to the bitumen surface, WCO molecules are distributed parallel to the bitumen surface, and SLRO morphology is different. Therefore, WWO molecules are more likely to penetrate the bitumen molecules, while WCO molecules are difficult to penetrate, while SLRO molecules are easy to penetrate the bitumen intermolecular pores due to their short chains. As a result, the permeability of WWO and SLRO is better than that of WCO.
(8) From the perspective of wettability, WCO has the strongest wettability, which is more suitable for aged bitumen regeneration. However, from the perspective of permeability, WCO has the weakest permeability among the three bio-oils, struggles to permeate the aged bitumen and is not conducive to the regeneration of aged bitumen. WWO has superior permeability and molecular activation ability, which is more suitable for the regeneration of aged bitumen. In view of this, WCO and WWO can be modified in further research to achieve greater suitability for aged bitumen regeneration. In addition, it is necessary to further study the permeation mechanism of the three kinds of bio-oil and deeply explain the internal reasons driving the difference of permeation.
(9) This study can provide a theoretical basis for the selection of reasonable rejuvenators for bitumen recycling engineering. At present, bio-oil has a wide range of sources and different qualities. Whether it can be used as a qualified rejuvenator needs to be considered. A qualified rejuvenator should not only have the ability to depolymerize asphaltene nanoclusters in aged bitumen, but also good diffusion performance; that is, wettability and permeability. As studied in this paper, rejuvenators with good wettability may not have good permeability. Therefore, they should be identified in practical projects. At the same time, research can provide theoretical support for the research and development of biomass rejuvenators.

## Figures and Tables

**Table 1:** Contact angles measured by MD simulation and laboratory experiment at 408.15 K.

Bitumen	WCO		WWO		SLRO	
	MD	Test	MD	Test	MD	Test
Virgin bitumen	24°	25.8°	50.5°	51.6°	61.5°	62.9°
RTFOT 85 min	29.5°	30.5°	53°	53.3°	65°	64.9°
RTFOT 180 min	31°	32.6°	54.5°	55.6°	67°	66.8°
RTFOT 360 min	36°	34.2°	58.5°	57.3°	69.5°	69.3°
RTFOT 720 min	40.5°	38.1°	61.8°	58.3°	71.5°	70°

**Table 2:** Basic Properties of the virgin bitumen.

Properties	Temperature	Results
Penetration (0.1 mm)	25 °C	82.5
Ductility (cm)	15 °C	122
Softening point (°C)		47.8

**Table 3:** Molecular compositions in virgin bitumen and RTFOT 85 min.

Bitumen Components		Virgin Bitumen		RTFOT 85 min	
		Weight (%)	No.	Weight (%)	No.
Asphaltene	Phenol	14.6	5	16.1	7
	Pyrrole		3		4
	thiophene		6		7
Aromatic	PHPN	44.8	39	39.4	30
	DOCHN		46		34
Saturate	Squalane	16.5	15	16.0	14
	Hopane		15		14
Resin	Quinolinohopane	24.1	4	28.5	3
	Thioisorenieratane		4		3
	Trimethylbenzeneoxane		29		37
	pyridinohopane		4		3
	Benzobisbenzothiophene		6		7

**Table 4:** Molecular compositions in RTFOT 180 min, RTFOT 360 min and RTFOT 720 min.

Bitumen Components		RTFOT 180 min		RTFOT 360 min		RTFOT 720 min	
		Weight (%)	No.	Weight (%)	No.	Weight (%)	No.
Asphaltene	Phenol	16.9	6	18.3	6	23.1	8
	Pyrrole		4		5		6
	thiophene		6		6		8
Aromatic	PHPN	37.3	30	32.7	26	30.3	24
	DOCHN		34		30		28
Saturate	Squalane	16.2	13	16.1	13	12.8	11
	Hopane		16		16		12
Resin	Quinolinohopane	29.6	4	32.9	4	33.8	5
	Thioisorenieratane		4		4		5
	Trimethylbenzeneoxane		34		38		39
	pyridinohopane		4		4		5
	Benzobisbenzothiophene		8		9		9

## Data Availability

Not applicable.
